# Molecular imaging nanoprobes and their applications in atherosclerosis diagnosis

**DOI:** 10.7150/thno.96037

**Published:** 2024-08-12

**Authors:** Huari Kou, Hu Yang

**Affiliations:** Linda and Bipin Doshi Department of Chemical and Biochemical Engineering, Missouri University of Science and Technology, Rolla, MO 65409, United States.

**Keywords:** atherosclerosis, molecular imaging, computed tomography, positron emission tomography, magnetic resonance imaging, photoacoustic imaging, ultrasound imaging

## Abstract

Molecular imaging has undergone significant development in recent years for its excellent ability to image and quantify biologic processes at cellular and molecular levels. Its application is of significance in cardiovascular diseases, particularly in diagnosing them at early stages. Atherosclerosis is a complex, chronic, and progressive disease that can lead to serious consequences such as heart strokes or infarctions. Attempts have been made to detect atherosclerosis with molecular imaging modalities. Not only do imaging modalities develop rapidly, but research of relevant nanomaterials as imaging probes has also been increasingly studied in recent years. This review focuses on the latest developments in the design and synthesis of probes that can be utilized in computed tomography, positron emission tomography, magnetic resonance imaging, ultrasound imaging, photoacoustic imaging and combined modalities. The challenges and future developments of nanomaterials for molecular imaging modalities are also discussed.

## 1. Introduction

**Figure 1:**
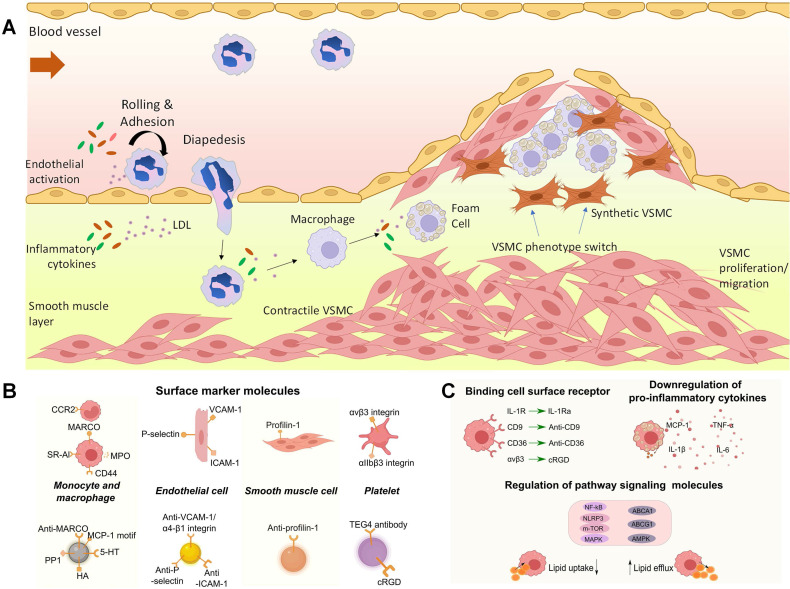
(**A**) The muscular artery and the cell changes that occur during atherosclerosis progression to thrombosis. (**B**) Surface marker molecules and (**C**) cell surface receptor and regulate atherosclerosis pathology on the molecular basis by influencing signaling molecules inside (pathway proteins) or outside (cytokines) cells. (**A**) adapted with permission from 3 copyright 2022, Portland Press. (**B**) and (**C**) adapted with permission from 6 copyright 2023, Elsevier.

Atherosclerosis is a lipoprotein-driven inflammatory disease, which is characterized by plaque formation in the arterial tree. The slow progress of atherosclerosis usually takes several decades until plaque rupture and thrombi formation happen, leading to serious consequences such as strokes or infarctions 1, 2. In general, atherosclerosis lesion development involves three stages (Figure 1 A). In normal artery, leukocytes can easily pass through the vascular without attaching to endothelial monolayer. However, in the initial stage of atherosclerosis, dysfunctional endothelial cells express adhesion molecules and capture passing leukocytes. Meanwhile, leukocytes migrate into the intima and mature into macrophages with the change of endothelial permeability and composition. The increase of arterial permeability will cause the deposition and penetration of cholesterol enriched low-density lipoprotein (LDL). In this stage, several cytokines are involved. For example, the vascular cell adhesion molecule-1 (VCAM-1) from endothelial cells and integrin α4β1 expressed on leukocytes will be widely secreted and enhance leukocyte adhesion. And the chemoattractant protein-1 (MCP-1/CCL2) can promote the migration of monocytes by binding to C-C chemokine receptor type 2 (CCR2) on monocyte surface. Then, local macrophage colony-stimulating factors (M-CSF) will stimulate the monocyte differentiate into macrophage with the cytokine concentration increase like tumor necrosis factor (TNF) and interferon-γ (IFN-γ). The accumulated macrophages will uptake lipid and yield foam cell formation a process in which the expressing of scavenger receptors (SRs) such as SR-A, CD36, and CD68 plays an important role. Next, smooth muscle cell migrates from the media to the intima. And the synthesis of extracellular matrix macromolecules will also increase. Foam cell apoptosis causes extracellular lipid accumulation and forms a lipid-rich pool called plaque necrotic core. The last stage of atherosclerosis is thrombosis. Thrombi usually arise after the physical disruption of plaque, leading to interruption of local blood flow or embolization in distal arteries 3-5. In this regard, it is essential to detect high-risk or vulnerable plaque for atherosclerosis management. Macrophages play a critical role in the progress of plaque lesion, such as the uptake and metabolism of lipoproteins and secretion of growth factors, contributing to plaque development. The identification of dysfunctional endothelial cells and recruited macrophages enables the diagnosis of plaques and allows for therapeutic interventions to modify the development of atherosclerosis. Furthermore, due to various cytokines are involved, these cytokines can act as targeting molecules in atherosclerosis studies, which are displayed in Figure 1B and C 6.

Medical imaging technologies have evolved from structure imaging to functional imaging and molecular imaging 7. Molecular imaging can visualize and quantify biological processes occurring at cellular and molecular levels. The realization of molecular imaging benefits from the application of highly specific and sensitive probes as well as high-resolution imaging instruments. Compared with conventional diagnostic imaging methods, probes are usually utilized to detect diseases in their early stages by monitoring molecular abnormalities rather than imaging the final effects of molecular alterations, enabling personalized and early-stage diagnosis and treatments. As one of the crucial components in molecular imaging, nanoparticles have been intensively studied for drug delivery and diagnosis for their molecular targeting effects 8-10. And it has been proved that optimized nanoparticles can significantly improve agent accumulations in targeting lesions, which can enhance diagnosis and therapy. And several nanomaterial systems are studied including metallic nanoparticles 11, 12, amphiphilic polymers 13-15, solid core polymer nanoparticles and lipid-based nanoparticles 16-18. Owing to the unique properties conferred by their size, functionalization abilities and modular structure, biomedical nanoparticles have continuously been exploited and used in the field of medical imaging. Meanwhile, molecular imaging is usually conducted in a noninvasive way, thus decreasing patient complications, such as wound infections, thrombosis formation, and artery rupture 19. Imaging modalities have been developed and applied to cardiovascular diseases like computed tomography (CT), positron emission tomography (PET), magnetic resonance imaging (MRI), single-photon emission computed tomography (SPECT), ultrasound, photoacoustic imaging (PAI). These modalities have been widely used in pre-clinical and clinical studies. In this review, we will summarize and discuss the recent developments of molecular imaging methods for atherosclerosis diagnosis.

## 2. Computed Tomography (CT)

CT is a molecular imaging method that combines both X-ray and computer technology. Basic CT equipment usually includes three components: X-ray source, X-ray detector and sample stage. The selection of X-ray source energy needs to be optimized based on sample conditions. Too high energy will decrease the attenuation time, causing a poor contrast. On the other hand, too low energy will limit the penetration distance, causing weak signal. The sample size should also be taken into consideration. Generally, a larger size of the sample requires higher X-ray energy. As a result, nano CT works in energy ranges 5-30 keV. Clinical scanners work from 80 to 140 keV, while heavy industrial scanner needs much higher energy of > 400 keV. When an X-ray beam traverses an object, the original beam will be absorbed or deflected. X-ray detector records the spatial pattern of transmitted X-ray and corresponding intensities. When considering the interaction between X-ray and exposed objects, two effects are included in the imaging process, namely, photoelectric and Compton effects. Photoelectric effect indicates an electron ejection from the most inner electron shell of an atom (the K shell) when the atom is irradiated by electromagnetic radiation. When the incoming photon has higher energy than the binding energy of K shell electron, it is absorbed and excites K shell electron ejection. This effect is element specific for the specific binding energy of different elements. The Compton effect is the effect between incoming photons with valence electrons, which is less relevant to the atomic number. The collision of photon and electron causes the valence electron ejection and X-ray scattering 20. These effects will weaken the beam intensity called X-ray attenuation. These composition related properties make CT can be used for human body imaging. When X-ray passes through a human body, different tissues show different attenuation capabilities. It would give detailed information on the whole body, including bones, muscles, organs, and vessels. CT imaging can be conducted with or without contrast reagents. Usually, the contrast is high enough to distinguish between bone and its surrounding tissues. However, it becomes difficult to define soft tissues, like tumors and normal organs. In this situation, contrast agents, which are usually injected or taken orally, can be a choice to elevate imaging quality for organs and tissues. As a rule of thumb, contrast materials possessing higher density or high atomic number tend to show better X-rays attenuation. Consequently, X-ray attenuating contrast media containing atoms of high atomic number (most commonly iodine or barium), are frequently used in clinical settings to obtain images of soft tissues.

### 2.1 Iodine based contrasts

An ideal contrast should possess properties including good stability, low toxicity, and high X-ray attenuation coefficients. For its capability to stop X-rays, iodine and its derivatives are commonly used in clinical practices. 1,3,5-triiodobenzene and its derivatives are the main commercialized compounds for CT, such as iohexol (trade name Omnipaque, GE Healthcare, USA), iopromide (trade name Ultravist, Bayer Healthcare, Germany), and iodixanol (trade name Visipaque, GE Healthcare) 21. However, iodine compounds have limitations such as short imaging time, lacking specificity, and causing renal toxicity. The rapid wash-in and wash-out of iodine-based small molecule contrasts makes it hard to achieve a long circulation time 22, 23.

Developing contrast agents with multifunction and high sensitivity can be a good approach to improve CT imaging accuracy and overcome conventional contrast shortcomings. Ghaghada *et al.* applied liposomal-iodine nanoparticle for atherosclerosis early detection. The nanoparticle was injected 4 days before the CT imaging allowing a sufficient accumulation of nanoparticles in abnormalities and clearance in systemic circulation 24. This nanoparticle enhanced CT shows high sensitivity to detect aortic wall degeneration and inflammation, which is associated with endothelial dysfunction and increased endothelial permeability. Kee *et al.* designed phosphatidylserine (PS) decorated liposomal contrast (iodixanol) for macrophage imaging by CT 25. It is found PS contained liposome with a proper size about 112 nm shows an enhanced cell uptake by macrophage. And the cell uptake can also be significantly improved by increasing PS ratio, which indicating an effective component for macrophage targeting. PS is externalized on the cell surface of senescent cells and is a potent target for macrophage recognition and their subsequent uptake. In addition, a faster clearance of PS liposome is confirmed compared with dipalmitoyl phosphatidylcholine (DPPC) nanoparticles with same sizes.

### 2.2 Gold based contrasts

**Figure 2:**
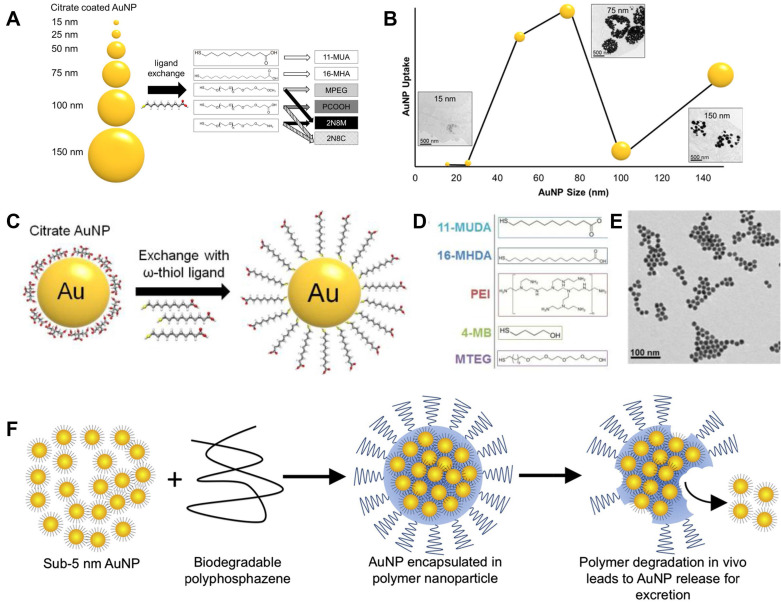
(**A**) Illustration of the range of gold nanoparticle sizes and chemical structures of coating ligands; (**B**) Gold uptake in monocytes incubated with different gold nanoparticles. (**C**) Schematic of ligand exchange for citrate capped gold nanoparticles; (**D**) Chemical structures of 5 formulations further used in experiments; (**E**) TEM image of 11-MUDA capped gold nanoparticle. (**F**) Illustration of biodegradable gold nanoparticles. (**A**) and (**B**) adapted with permission from 28 copyright 2017, American Chemical Society. (**C**-**E**) adapted with permission from 29 copyright 2016, Elsevier. (**F**) adapted with permission from 31 copyright 2016, Elsevier.

Besides iodine compounds, some other compounds and nanomaterials contain Au, Bi, Ta, etc. display good X-ray attenuation capabilities and are viewed as promising CT contrasts, which can help to decrease the patient exposure to radiation and increase image quality. Among these potential contrasts, gold nanoparticle has been widely studied and reported for its good chemical stability, low toxicity, and high X-ray attenuation coefficient. Besides the common advantages, gold nanoparticle also exhibits good penetration properties passing through underlying tissues 26, 27. Chhour *et al.* studied the size and coating layer effects on monocyte uptake and CT imaging 28. As shown in Figure 2A, gold nanoparticles with sizes from 15 nm to 150 nm were obtained in their studies. Meanwhile, the coating layers were also studied including distal carboxylic acids, poly(ethylene-glycol) with methoxy (MPEG), carboxylic acid (PCOOH), and amine functional groups. Based on the gold concentrations (Figure 2B), except MPEG, highest uptake amounts were achieved by the nanoparticles with sizes of 50 and 75 nm ignoring coating layer. These nanoparticles give much higher attenuations than 100 and 150 nm nanoparticles after nanoparticle treatment range from 0.1 to 0.5 mg/mL. And this phenomenon was also confirmed by inductively coupled plasma optical emission spectroscopy (ICP-OES) results, when the gold nanoparticles coated with PCOOH was selected for CT testing. Gold nanoparticles with different ligands are used to label monocytes for monitoring monocyte recruitment and revealing atherosclerosis developments. Citrate capped gold nanoparticle is chosen for ligand exchange in this study (Figure 2C). To minimize the effects of nanoparticles to monocytes and maximize cell uptake, cell viability and cytokine release were studied for various ligands (Figure 2D) including 11-mercaptoundecanoic acid (11-MUDA), 16-mecaptohexadecanoic acid (16-MHDA), poly(ethyleneimine) (PEI), 4-mercapto-1- butanol (4-MB), and 11-mercaptoundecyl-tetra (ethylene glycol) (MTEG). 11-MUDA was characterized by transmission electron microscopy (TEM) (Figure 2E) and selected to track monocyte recruitment in atherosclerosis with CT for its good biocompatibility and efficient cell uptake. A continual attenuation increase is observed in the aortas from mice receiving gold nanoparticle labeled monocytes, while the other mouse groups do not show significant increases within 5 days. This study provides a feasible way of tracking and quantifying monocyte recruitment with CT imaging 29.

The excellent chemical stability makes gold an ideal contrast for CT. Nevertheless, it also creates concerns for its practical applications. Nanoparticles with diameters larger than 5.5 nm would have a long blood circulation, while those smaller than 5.5 nm could be easily excreted by kidney 30. The application of gold nanoparticles larger than 5.5 nm makes a concern for its long-term retention in body and side effects for subsequent imaging. To address this problem, Cheheltani *et al.* designed a gold nanoparticle containing gold nanocores and biodegradable poly di(carboxylatophenoxy)phosphazene (PCPP) coating layer 31. As illustrated in Figure 2F, this Au-PCPP nanoparticle showed a diameter of about 100 nm, while gold nanocores are smaller than 5 nm. The nanoparticle size can be tuned by varying the ratio of polyethylene glycol-polylysine (PEG-PLL) in final formulation. TEM image verifies the nanoparticle degradation and nanocore release after incubated with 10% serum for 7 days. And the gold release speed is related to Au-PCPP nanoparticle size. Smaller nanoparticle size can accelerate the degradation speed, which may be due to the specific surface area increase. Their results show that over 60% gold nanoparticles are released after 8 days incubation with cells.

### 2.3 Metal alloy based contrasts

Bimetallic nanoparticles are also studied by Chu *et al.* for CT imaging 32. Bovine serum albumin (BSA) is used as a template to direct the formation of Au-Ag nanoparticles. Au-Ag alloy nanoparticle exhibits a neat X-ray absorbance comparing with pure Au or Ag nanoparticles, which promises an increase of CT image brightness. And the optimized Au-Ag nanoparticle with a ratio of 60% gold shows 1.8 times attenuation than Iohexol at a concentration of 80 mM. Guo *et al.* synthesized 2D Pd and Au nanomaterial with folic acid to target activated macrophages in atherosclerotic plaque 33. Compared with normal aorta, enhanced signals were detected *in vivo* with CT in advanced atherosclerotic plaques. Blocking studies are also conducted to verify the targeting effects. Excessive folic acid pre-injection can significantly decrease the CT signal of Pd and Au nanomaterial. This nanoprobe is also applied in PAI and SPECT imaging and gives enhanced signals in atherosclerotic plaques.

The utilization of nanomaterials as CT contrasts can help to get enhanced images and detect atherosclerotic lesion. It is of importance to design nanoprobe with good X-ray attenuation capability and specificity to atherosclerosis plaque. As summarized in Table 1, the research of CT contrasts is mainly focused on gold nanoparticles for its excellent stability and X-ray attenuation capability. However, the discussed research also shows some alloys can further improve the imaging quality. It indicates that CT contrast design for atherosclerotic diagnosis can also extend to some other elements, like Bi, Ta, Ba and Pt. For example, Bi has better X-ray attenuation property than gold. And the disadvantages of Bi can be addressed with proper design and synthesis methods 34-36. Ta_2_O_5_ can also be used as CT contrast for its good chemical stability and biocompatibility 37, 38. Morphology control can be another consideration for CT contrast design including size and shape. It has been widely studied that the nanoparticle size can significantly influence the metabolization or interaction with cells and organs. Interactions between silver nanoparticles and bacteria are reported by Kim *et al.* 39. It is found that small plate-shaped nanoparticles show better antibacterial effects than cubic and spherical nanoparticles with larger size. It is observed that gold nanoparticle size can influence histological alterations in the rat liver 40. Consequently, the CT image may also be improved by tuning contrast morphology.

## 3. Positron Emission Tomography (PET)

It has been five decades since the practical application of the first PET scanner in 1975 45. Although it has been verified as a powerful tool in biology and physiology studies, its application in clinical field is severely hindered for its limited availability and high cost. This situation is improving now resulting in the increase of a clinical need for more accurate and specific diagnostic techniques. And the increase of on-site cyclotrons also makes PET more achievable for both laboratory and clinical study 46. The basic PET principle can be described as follows: A positron is emitted from the nucleus with a neutrino in β decay. And this positron randomly travels until it annihilates with an electron and gives two 511 keV photons in opposite directions. The PET scanner ring is designed to detect these photons as coincidences. Advanced PET scanner can get 3D images, by detecting coincidences in all possible directions rather than coincidences only in the same axis rings. To get better resolution, several imaging reconstruction models have been developed such as static, gated, and dynamic image construction 47.

### 3.1 ^18^F labeled nano probes

The radioactive isotope plays a crucial role in PET process. Among the FDA approved PET tracers, ^18^F-fluorodeoxyglucose (^18^F-FDG) and sodium ^18^F-fluoride (^18^F-NaF) are the most used probes for atherosclerosis detection. ^18^F-FDG can be easily taken up by macrophages in the plaque via glucose transporter. And NaF can interact with the hydroxyl group in hydroxyapatite at the sites of active calcification/ossification and no other organs or disease processes. Consequently, PET imaging with ^18^F-FDG and ^18^F-NaF can assess atherosclerotic disease at molecular level. Shen *et al.* studied the early detection of atherosclerosis with ^18^F‑FDG in Wistar rat model. Their results show that the uptake of ^18^F-FDG in high-fat-diet group is much higher than in normal diet group in both iliac artery and abdominal aorta 48. However, the uptake of^ 18^F-FDG can be easily affected by several factors like hypoxia and increased myocardial muscle activity. The coronary and cerebral arterial segments cannot be well evaluated for the high-level glucose uptake in heart and brain. And in some research, it does not show significant differences between patients with and without atherosclerosis 49. The short half-life time also hinders ^18^F-FDG application in atherosclerosis diagnosis. It cannot accumulate enough FDG in the lesion before the signal becomes too low for detection 50, 51.

**Figure 3:**
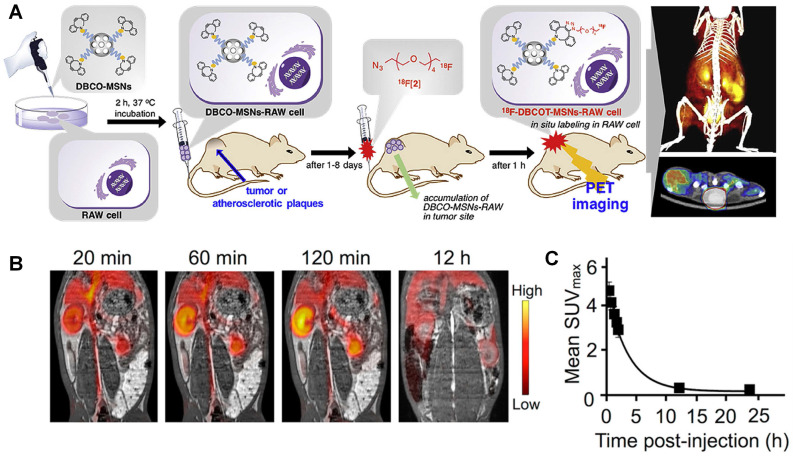
(**A**) Schematic procedure for the in-situ synthesis of ^18^F-labeled aza-dibenzocycloocta-triazolic mesoporous silica nanoparticles into RAW 264.7 macrophage cells in a living specimen for the cell tracking PET imaging. (**B**) Coronal PET/MRI fusion images of an atherosclerotic rabbit; (**C**) Clearance kinetics of ^89^Zr-HA-NPs determined noninvasively in rabbits by measuring SUV in the aortic blood. (**A**) adapted with permission from 52 copyright 2019, Elsevier. (**B**) and (**C**) adapted with permission from 59 copyright 2017, American Chemical Society.

Jeong *et al.* designed ^18^F labelled silica nanoparticles for PET imaging 52. Mesoporous silica nanoparticle exhibits good biocompatibilities and low cytotoxicity especially after modified with PEG. Meanwhile, mesoporous silica nanoparticle has high capability for drug loading, which makes a potential option for drug delivery system. Macrophages can be labeled with ^18^F after incubation with this silica nanoparticle. Then these macrophages are used to track tumor issue and atherosclerotic plaques. As mentioned before, the short half-life of ^18^F (t_1/2_=109.8 mins) makes it difficult to achieve a long-term monitoring for the injected macrophages. This problem can be overcome with the help of strain-promoted alkyne azide cycloaddition (SPAAC). Briefly, both functionalized silica nanoparticle and ^18^F compound are designed as SPAAC reactants. As shown in Figure 3A, firstly, silica nanoparticle labelled macrophages are injected into mouse model. After 1-8 days, these labelled macrophages will accumulate in tumors or atherosclerotic plaques. Then, the ^18^F compound can be injected and trigger SPAAC reaction, when ^18^F compounds reach silica nanoparticles concentrated areas. This design makes it possible to monitor labelled nanoparticles until these nanoparticles are metabolized. And it also provides a new thought to hire short half-life time isotopes for chronic diseases.

### 3.2 Other isotope labeled nano probes

Kim *et al.* designed mannose labelled nanoparticle to improve its PET imaging capability 53. Compared with glucose, mannose can be uptake via glucose transporter, and bonded to mannose receptors. This ^68^Ga-NOTA-MSA nanoparticle is synthesized by conjecting 2-(p-isothiocyanatobenzyl)-1,4,7-triazacyclononane-1,4,7-triacetic acid (SCN-NOTA) and Neomannosylated human serum albumin (MSA). ^68^Ga-NOTA-MSA shows higher maximal standardized uptake values (SUVs) than ^18^F-FDG probe in both abdominal aorta and thoracic aorta. However, both probes cannot give desirable results by comparing the signals for inferior vena cava in atherosclerosis and control groups. Nie *et al.* labelled diacetyl-bis (N^4^-methylthiosemicarbazone) with ^64^Cu (^64^Cu-ATSM) to target hypoxia areas, which has been identified as a potential factor in the formation of vulnerable plaque 54-56. And this lipophilic molecular shows good cell membrane permeability. Furthermore, Cu(Ⅱ) can be reduced by ubiquinone oxidoreductase and trapped in hypoxic cells. Compared with ^18^F-FDG, it shows a higher cellular uptake and a shorter blood retention time, which can provide higher contrast and accelerate PET imaging process. ^64^Cu-ATSM displays stable radioactivity and significant difference between ApoE^-/-^ and control group from 7.5 mins post injection, while the signal from ^18^F-FDG keeps increasing until the end of imaging (60 mins) 57.

The rapid blood clearance of hyaluronan (HA) makes it a potential biodegradable material for drug delivery. Studies show HA with low molecular weight can stimulate inflammation, while the highly polymerized HA can suppress this process. During inflammation, the hyaluronan lining on vascular endothelium mediates immune cell rolling and extravasation, and at the site of inflammation the hyaluronic-rich microenvironment stimulates the tissue penetration by and division of immune cells. And the hyaluronan-CD44 receptor interactions play a key role in tumor progression 58. Beldman *et al.* designed HA nanoparticles (HA-NPs) by reacting amine-functionalized oligomeric HA with ^89^Zr labelled cholinic ester 59. The aortic macrophage showed much higher uptake of HA-NPs than surrounded normal tissue macrophages. Representative PET/MRI fusion images are displayed in Figure 3B, on which the circulation kinetics is also monitored (Figure 3C). Quantified SUV values are about 4 times higher in aorta than muscles at 12 h post injection. However, compared with 6-week high-fat diet group, 12-week high-fat diet group shows much lower HA-NP concentration in aorta. They suggested it may be due to the cell apoptosis and necrosis. The HA-NPs treatment also exhibits therapy effects to atherosclerosis development due to the interaction of HA-NP and immune cells. Studies show that HA plays a vasoprotective role in the healthy vascular glycocalyx that inhibits platelet adhesion and leukocyte rolling 60. Besides HA, chitosan is another widely applied natural polymer in clinical research for its good biocompatibility and biodegradability 61-63. ^89^Zr labeled chitosan nanoparticle was synthesized for inflammation imaging by Fairclough *et al.* To synthesize chitosan-based nanoparticles, polyanion pentasodium tripolyphosphate (TPP) was used to crosslink linear chitosan polymer. Then chitosan nanoparticle was labelled with ^89^Zr by incubating chitosan nanoparticle with ^89^Zr-oxalate for 45 mins. And the nanoparticle size can be controlled from 343 to 545 nm by adjusting the ratio of chitosan and TPP 64.

As summarized in Table 2, various matrix materials have been utilized in the design and synthesis of PET contrast for cardiovascular disease, especially for atherosclerosis. But the options of radioactive isotopes are still limited for the requirements of proper half-life time, even though these isotopes can label various nanoparticles by incubation with different matrixes. And only a trace amount of isotope is needed for excess precursors, which makes it more efficient to achieve a high labeling yield. Noticeably, due to the half-life time of radioactive isotopes, fast and simple procedures are usually preferred in PET probe design and synthesis. PET imaging is limited by lack of anatomic information when it offers high sensitivity and imaging depth. PET shows limited spatial resolution in detecting submillimeter lesions in arterial structures, which is one of the main challenges in disease diagnosis. Especially, the plaque size is only about several hundred micrometers at the early stage of atherosclerosis. Furthermore, the respiratory and cardiac motion leads to more difficulties in data acquiring process. As a result, PET are usually combined with CT which will be discussed later.

## 4. Magnetic Resonance Imaging (MRI)

Different from CT and PET, no X-ray or ionizing radiation are involved in MRI. Meanwhile, MR signal can be produced without any contrasts making it preferred in pre-clinical and clinical research. The mechanism can be briefly described as following: When the water molecule is exposed to an external magnetic field, the hydrogen nuclear spins of the protons will equilibrate with a frequency determined by the strength of the magnetic field. Then, the applied magnetic field will flip protons to transverse to the external field from parallel. Those protons will flip back to equilibrium once the field is removed. Along with the spin-lattice/longitudinal relaxation (T1) and spin-spin/transverse (T2) relaxation, different proton densities give different contrasts in a magnetic resonance image 79. Two different types of images can be obtained by detecting T1 or T2 relaxation. Generally, T1-weight image is useful to assess the cerebral cortex and fatty issue, while T2-weight image can help to reveal the inflammation and assess zonal anatomy.

### 4.1 Gadolinium based contrasts

To enhance the difference between normal and abnormal tissues, MRI contrasts, especially gadolinium compounds, are widely utilized in practical applications. Gadolinium based contrasts show good paramagnetic properties due to the presence of 7 unpaired electrons in Gd(Ⅲ). Gd contrasts can shorten T1 and T2 relaxation time of neighboring water protons, which can increase the T1-weighted intensity and decrease the T2-weighted intensity 80. After Gd-diethylenetriamine-pentaacetic acid (Gd-DTPA) was induced as MRI contrast for diagnostic application in 1984, Gadolinium compounds have been commercialized and widely used in clinical applications like Gd-DTPA (Magnevist®), Gd-DTPA-BMA (OmniScan®), Gd-HP-DO3A (ProHance®) and Gd-DOTA (Dotarem®) 81.

**Figure 4:**
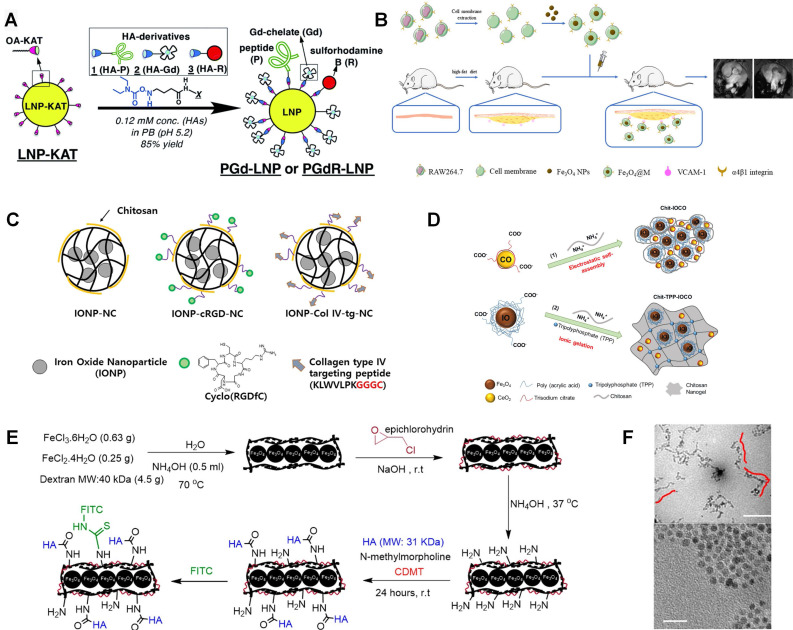
(**A**) Illustration for the preparation of multi-functionalized LNPs from KAT ligation labelled LNP-KAT nanoparticle. (**B**) Fe_3_O_4_@M as an MRI imaging contrast agent for detecting foam cells. (**C**) Schematic illustration of the prepared IONP loaded nano-carriers; pluronic based nano-carrier (IONP-NC), cyclo RGD peptide conjugated nanocarrier (IONP-cRGD-NC), and collagen type IV targeting peptide conjugated nano-carrier (IONP-Col IV-tg-NC). (**D**) Image Illustrating the Synthesis of Chit-IOCO and Chit-TPP-IOCO. (**E**) Illustration of HA-NWs synthesis procedure; (**F**) TEM images for HA-NWs showed the elongated shape of HA-NWs. Three representative worms are traced with red lines. (**A**) adapted with permission from 83 copyright 2020, The Royal Society of Chemistry. (**B**) adapted with permission from 87 copyright 2021, Elsevier. (**C**) adapted with permission from 88 copyright 2018, Elsevier. (**D**) adapted with permission from 93 copyright 2021, American Chemical Society. (**E**) and (**F**) adapted with permission from 94 copyright 2018, American Chemical Society.

However, these mentioned gadolinium compounds are usually used as non-specific extracellular tracer. The plaque size, especially for early-stage atherosclerosis, is usually submillimeter, which requires a good targeting ability to get effective diagnosis. To target atherosclerotic plaque, Li *et al.* designed class AI scavenger receptors (SR-AI) targeted nanoparticles in which PP1 (16-mer peptide, LSLERFLRCWSDAPAK) acts as a targeting molecular 82. GdCl_3_ and HAuCl_4_ are mixed with Glutathione (GSH) solution. Then, NaOH is added to trigger the reduction of GSH with gold and gadolinium. PP1 peptide is conjugated with the nanoparticle by mixing with it in 1-ethyl-3-(3-dimethylaminopropyl)carbodiimide hydrochloride (EDC) solution. Both nanoparticles with and without PP1 peptides show much higher signal intensities than Magnevist at the same Gd^3+^ concentration. They indicated it may be attributed to the longer rotational correlation time caused by confined Gd^3+^ tumbling in biomacromolecule. The potential to detect macrophages is also verified by incubation with different formulations. Targeting nanoparticles gave the highest intensity compared with the nontargeting nanoparticles and competitive inhibition. Fracassi *et al.* developed LDL-mimetic lipid nanoparticles for MRI, which was labelled with Gd and sulforhodamine B 83. And the nanoparticle synthesis method is illustrated in Figure 4A. Lipid nanoparticle is functionalized with amide-forming potassium acyltrifluoroborate, which makes it can react with hydroxylamine derivatives of apolipoprotein-mimetic peptide and Gd(Ⅲ)-chelate. Meanwhile, sulforhodamine B is also utilized in this nanoparticle for fluorescent imaging. Similarly, Yoo *et al.* designed amphiphile micelles for atherosclerotic MRI imaging. This micelle contains gadolinium chelator DTPA, DTPA-bis(stearylamide) (Gd), and 1, 2-distearoyl-*sn*-glycero-3-phosphoethanolamine-N-[(poly(ethylene glycol)-2000]-DTPA(Gd) (DSPE-PEG2000-DTPA(Gd)). Cysteine-arginine-glutamic acid-lysine-alanine (CREKA) is incorporated for targeting to clots leaded by the unstable plaque rupture 84. Well functionalized lipid nanoparticle can be a good platform for Gd labelling.

### 4.2 Iron oxide based contrasts

Iron oxide can represent another type of MRI contrast, namely, superparamagnetic magnetite. And iron oxides can reduce the T2 signal of tissues containing iron oxides. In addition, compared with Ga based contrast, iron oxide can decrease the Ga induced nephrogenic systemic fibrosis to patients with renal diseases. Tarin *et al.* designed gold-coated iron oxide nanoparticles (IONPs) for MRI, which was also labeled with anti-CD163 antibody to image macrophages in atherosclerosis plaque 85. The gold coating layer can effectively protect the magnetic core from degradation and allow for versatile functionalization by using thiol-ending ligands. The nanoparticle core is gold coated Fe_3_O_4_, which is covered with thiol ligands bearing either a mannose or a carboxylic acid. A silica coating layer can be another option for iron nanoparticle protection and modification, which can also promote material biocompatibility 86. As shown in Figure 4B, macrophage membrane coated Fe_3_O_4_ nanoparticle is also studied as MRI contrast to detect early plaque for its specific recognition of integrin of α4β1 to VCAM-1. And α4β1 integrin is over expressed on macrophage membrane, like RAW 264.7, which can be used to prepare cell membrane solution. Cell membrane coated Fe_3_O_4_ nanoparticles can be easily synthesized by mixing membrane and Fe_3_O_4_ nanoparticle and followed magnetic separation 87.

In the research from Kim *et al.*, targeting effects of Arg-Gly-Asp (RGD) peptide and collagen type IV targeting peptide (Col Ⅳ-tg-peptide, Sequence: KLWVLPKGGGC) were compared via MRI method 88. As shown in Figure 4C, the iron oxide loaded chitosan nanoparticle is chosen as a basic no targeting carrier. Then, targeting nanocarriers are synthesized by conjugating cRGD or Col Ⅳ-tg-peptide to nanoparticle via cross-linker Poly(ethylene glycol) (N-hydroxysuccinimide 5-pentanoate) ether N'-(3-maleimidopropionyl)aminoethane (Mal-PEG-NHS). It is found that RGD peptide can bind to the α_v_β_3_ integrin receptor, which is associated with angiogenesis over expressed in the vascular endothelial cells and macrophages. And Col Ⅳ-tg-peptide can also be used to target collagen type Ⅳ abandoned existing on blood vessels. Especially, it can be easily exposed in atherosclerotic lesion with the enhancement of tissue permeability. Their research shows that the RGD peptide displays about twice signal enhancement than Col Ⅳ-tg-peptide. Bonnet *et al.* reported human scFv-Fc antibody functionalized superparamagnetic iron oxide nanoparticle for atherosclerosis MRI imaging, which can recognize overexpressed galectin 3 by the TREM2-positive foamy macrophage subset in atherosclerosis. This nanoparticle was also utilized as nanocarrier for alpha-tocopherol drug delivery in this research 89. Similarly, Rapamycin is used as a model drug loaded on superparamagnetic nanoparticles in Zhang's studies. Rapamycin can be specifically delivered to vascular smooth muscle cells via Prodilin-1 antibody labelling, which triggers cardiovascular diseases 90. These theranostic nanoparticles can efficiently alleviate the progression of atherosclerosis.

Besides these clinical medicines, Yao *et al.* explored connective tissue growth factor (CTGF)-targeted ultrasmall superparamagnetic iron oxides for MRI within carotid atherosclerotic lesions. In comparison with lgG labelled nanoparticle, anti-CTGF labelled nanoparticle shows therapy benefits indicating a potential application in atherosclerosis treatments 91. As the *in vitro* research indicated, anti-CTGF can reverse vascular smooth muscle cell proliferation and migration, which can be accelerated by overexpressing CTGF in atherosclerotic lesion 92. Wu and his colleges combined Fe_3_O_4_ and CeO_2_ in theranostic nanoparticles, which can serve as both MRI contrast and reactive oxygen species (ROS) scavenger 93. Ce_2_O_3_ can react with and transfer superoxide radicals into oxygen and H_2_O_2_. And the Ce^3+^ can further react with H_2_O_2_ and produce H_2_O. In their study, poly (acrylic acid)-coated Fe_3_O_4_ and trisodium citrate coated cerium oxide are combined with chitosan in nanoparticles. As shown in Figure 4D, this nanococktail can be synthesized via both electrostatic self-assembling and ionic gelation. Polysaccharide HA coated Fe_3_O_4_ nanowire was also designed for MRI imaging, where the HA is used for CD44 targeting. As shown in Figure 4E, the Fe_3_O_4_ nanowire is synthesized in ammonia solution in the presence of dextran. And this nanowire is further conjugated with HA. TEM images in Figure 4F reveals the nanowire is constructed with nanoparticles. Their studies suggest that the HA-nanowire induced much less inflammatory than HA-nanoparticle by comparing the mRNA expression levels including IL-1α, IL-1β and MCP-1 94.

### 4.3 Other contrasts

Although Gd and Fe compounds have exhibited excellent MRI enhancement in relevant research, some other contrasts are also studied for atherosclerosis imaging. Sherin *et al.* designed curcumin incorporated TiO_2_ nanoparticles for MRI. And MCP-1 antibody is linked to the nanoparticles to target macrophage-foam cells in atherosclerotic plaques 95. MnFe_2_O_4_ is also utilized as MRI contrast in the design of theranostic nanoparticle for atherosclerosis, which shows excellent magnetic properties and enhanced sensitivity without obvious toxicity. MnFe_2_O_4_ is stabilized by Poly (lactic-co-glycolic acid) (PLGA) and labelled with anti-VEGFR-2 antibody to target the aortic endothelial cells. MFe_2_O_4_ represents a class of magnetic sensitive compound, where M= Mn, Fe, Co, Ni 96.

Generally, MRI contrasts can be classified into two groups. One is paramagnetic compounds mainly containing Gd compounds. And the other group contains iron, manganese, or other transition metal compounds. Chelation and some other natural polymers are usually induced in MRI contrasts which can reduce the toxicity of metal ions and sometimes act as targeting molecule. Besides studies mentioned above, various research has been done in recent years (Table 3). Several parameters should be considered in MRI probe design for atherosclerosis including: (1) materials with enhanced MR effect in high and ultrahigh fields, (2) good specificity for selective organs and tissues to achieve high concentration in required sites, (3) improvements of tolerance in body.

## 5. Ultrasound

Ultrasound based techniques are very popular in clinics for its characters like rapid, cost-efficient, noninvasive, free from ionizing radiation and repeatable. The mechanism can be briefly described as following: In ultrasound examination process, body is exposed to high frequency sound waves. Then the sound wave will be reflected after interaction with body and tissues, which will be detected and recorded by ultrasound probe. The ultrasound images are generated based on the calculation of echo time, intensity, and some other parameters. Many different types of ultrasound methods are developed from traditional bright (B)-mode ultrasound, contrast enhanced ultrasound (CEUS), elastography method, to invasive spectrum-intravascular ultrasound (IVUS), transesophageal echocardiography, and epiaortic ultrasound 126-128. Regarding atherosclerosis diagnosis, on B-mode ultrasound, plaques that contain a large lipid core appear echolucent, whereas plaque fibrosis and calcification tend to appear echogenic. Conventional B-mode ultrasound applications in detecting atherosclerosis are really limited for its insufficient testing depth and low precision. The change of intima-media thickness (IMT) is applied as a main parameter in ultrasound-based atherosclerosis diagnosis, which may be another concern. Cuspidi *et al.* suggest that C-IMT index is more sensitive for vascular alterations due to hypertension rather than atherosclerotic plaques 129. CEUS can much improve the image quality compared with conventional B-mode ultrasound by hiring nano bubbles as contrasts. In this review, we would like to focus on the design on CEUS nanoprobe design and application in ultrasound examinations.

Ultrasound contrast agents required in CEUS are usually gas-filled microbubble agents. In ultrasound examination, ultrasound contrasts can resonate with ultrasound wave and be destroyed by ultrasound wave leading to better image and providing quantifiable data 126. The use of microbubbles for this purpose is based on observations made by Gramiak and Shah, who noticed a signal enhancement of blood in the aorta after injection of mechanically agitated saline containing small air bubbles 130. In fact, the contrast medium is formed from microbubbles that contain a gaseous SF_6_ interior encapsulated by a phospholipid capsule. The hydrophilic external surface and hydrophobic interior surface make it sufficiently stable in blood and to oscillate. Moreover, a proper size about 2.5 µm leads to a good permeation to internal vascular and being too large to cross the endothelium, which means that these bubbles remain intravascular 128. Inspired by their work, numerous ultrasound contrasts are developed in recent years.

Modifying commercialized CEUS contrasts can be a straightforward way to facilitate the clinical applications. Yang *et al.* directly mixed IL-8 antibody with USphere™ Labeler microbubbles to deliver IL-8 antibody. And they studied the inflammatory response and plaque stability of microbubble delivered IL-8 monoclonal antibody. It is found that IL-8 antibody can neutralize IL8-mediated inflammation and increase the stability of plaque 131.

Emulsion method is commonly used in nano bubble fabrication, especially, oil-water-oil (double) emulsion. It also allows the drug loading to nano bubbles of both hydrophobic and hydrophilic drugs. Zhang *et al.* designed anti-VEGFR-2 functionalized nanobubbles as ultrasound contrast via ultrasonic emulsion method 132. This lipid-based nano bubble material is made from DPPC, PEG and 1,2-Dimyristoyls-sn-glycerol-3-phosphocholine (DMPC). And nanobubble will form with continuous SF_6_ purging under sonication. After anti-VEGFR-2 conjugation, active targeting ultrasound contrast can be obtained. Perfluorooctyl bromide (PFOB) is adopted as ultrasound contrast core in the study from Li *et al.*, which shows good acoustic stability and long half-life 133. PLA and PFOB is dissolved in dichloromethane and then mixed with polyvinyl alcohol solution. After emulsification by sonication, PEG and Osteopontin (OPN) antibody conjunctions are followed. OPN is one of the biomarkers for vascular smooth muscle cell phenotypic conversion and can be used as a targeting molecular.

**Figure 5:**
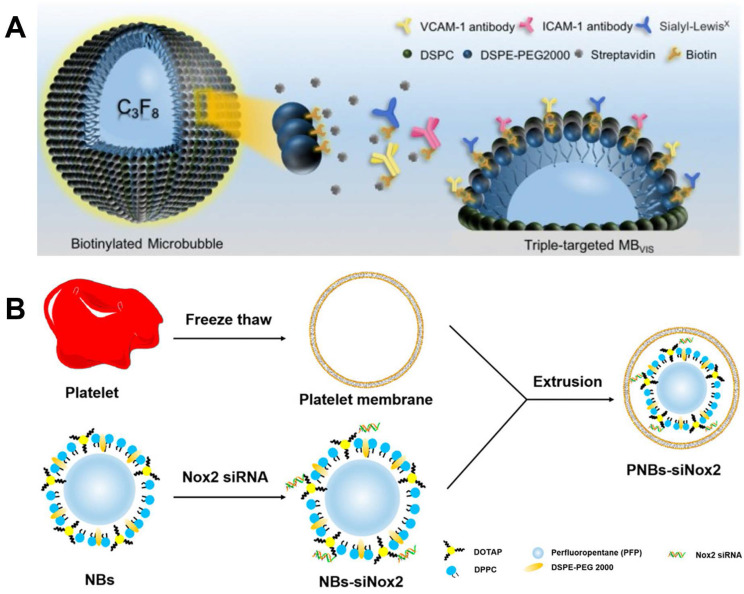
(**A**) Schematic diagram of targeted MB_VIS_. (**B**) Manufacturing of PNBs-siNox2. (**A**) adapted with permission from 135 copyright 2018, Ivyspring International Publisher. (**B**) adapted with permission from 138 copyright 2023, Elsevier.

As discussed before, the design of active targeting contrasts has been one of the most promising strategies for molecular imaging. Moccetti *et al.* compared the small peptide ligands affiliated endothelium-targeted microbubble contrasts including human P-selectin, VCAM-1, LOX-1 and von Willebrand factor (VWF). Both VCAM-1 and VWF show better image quality than the other two in mice and human patient plaque 134. As shown in Figure 5A, Yan *et al.* reported triple targeted microbubble contrast for ultrasound imaging to decrease off target effects. This leucocyte-like material is labelled with three targetings including VCAM-1/ICAM-1 antibodies and synthetic polymeric sialyl Lewis X mimicking leucocytes behaviors. The good affinity to inflammatory endothelium gives a significant signal increase compared to single and dual targeted contrasts 135.

Theranostic nanoparticle is also an important research direction for the controllable drug release. The drug release can be triggered with ultrasound wave by destroying gas core of the nanoparticle, which can further improve the drug uptake in lesion. Metha *et al.* synthesized theranostic 2-Hydroxypropyl-beta-cyclodextrin loaded nanoparticle 136. It showed an enhanced cellular uptake in murine cells. Yao *et al.* reported Sinoporphyrin sodium-mediated sonodynamic therapy for plaque neovascularization regression 96. Perfluoropentane cored nanoparticle is designed and stabilized with PLGA. Ramucirumab, an anti-VEGFR-2 antibody is used as a targeting molecular. This nanoparticle shows a good stability for over 7 days, which is a crucial parameter for nano bubble entrapped nanoparticles. It shows good inhibition to plaque neovascularization by inducing mitochondrial-caspase apoptosis in neovascular endothelial cells. Platelet membrane coated nanobubble contrast was reported and used for gene delivery by Hu *et al.* It has become increasingly apparent that, even at the outset, there is significant crosstalk between platelets and inflamed endothelium, which trigger autocrine and paracrine activation processes that lead to leukocyte recruitment into the vascular wall 137. As shown in Figure 5B, the nano bubbles are constructed with DPPC, 1,2-Dioleoyl-3-trimethylammonium-propane (DOTAP) and 1,2-distearoyl-sn-glycero-3-phosphoethanolamine-N-(methoxy(polyethyleneglycol)-2000) (DSPE-PEG) with a perfluoropentane core, while the platelet membrane is obtained via freeze thaw method. This well designed nanobubble can effectively target to collagen, foam cells and human umbilical vein endothelial cells. And benefit from the good targeting capabilities, an efficient gene delivery is also achieved 138.

As aforementioned, the emulsion and sonication methods are commonly used in nanobubble contrast design make the nanoparticle synthesis controllable and efficient. And volatile organic liquids are applied to form gas core. The contrast design focuses on the nanoparticle coating or ligand conjunctions. Similar with other modalities, various antibodies are hired as targeting molecules, which can specifically affiliate to different cell sites. Dual and triple targeting nano bubbles are also reported to improve targeting efficiency and decrease off-target effects.

And nano bubble contrasts show their unique advantage for drug and gene delivery. The hollow structure of nano bubbles makes it can be easily destroyed and trigger drug releasing. Furthermore, the fast clearance can also be accelerated with the ultrasound wave by degrading ultrasound contrasts. However, compared with other drug loading methods, it also shows relatively low drug loading ratio and efficiency.

## 6. Photoacoustic Imaging (PAI)

PAI method is developed based on photoacoustic effect and combines both optical contrast and acoustic detection technologies 139. When the biological tissue is exposed to laser pulses, electromagnetic waves will be absorbed and lead to a local temperature increase. The tissue expansion under heating causes ultrasound emission, which can be detected and reconstructed into images. Although the optical attenuation is improved compared with optical imaging, it is still limited to few centimeters in tissue when the resolution goes to submillimeter. And the detection of ultrasounds requires direct contact to tissue and suffers severe attenuation and phase distortion in bones. Many reports focus on the PAI applications in carotid atherosclerosis rather than coronary atherosclerosis due to the limited imaging depth. It is also the main reason that many atherosclerosis studies are currently limited to small animal models. The lasers used in PAI various from visible to region near infrared (NIR) range. Generally, NIR laser is preferred in practices for its relative better tissue penetrating capability up to several centimeters and the low absorption of tissue in this wavelength range 140-142.

Similar with MRI and CT, PAI can be conducted with or without external contrast. External contrasts are usually used to enhance signal response and improve image quality. Generally, these contrasts have their own fixed excitation wavelengths and increased penetration depths. A series of photoacoustic probes have been developed and reported in recent years. For the noninvasive imaging, these nano contrasts usually show deep tissue penetration and high spatial resolution, such as metal nanoparticles 143, quantum dots 144-147, small-molecule organic compounds 148-150, and semiconductive polymers 151-153.

**Figure 6:**
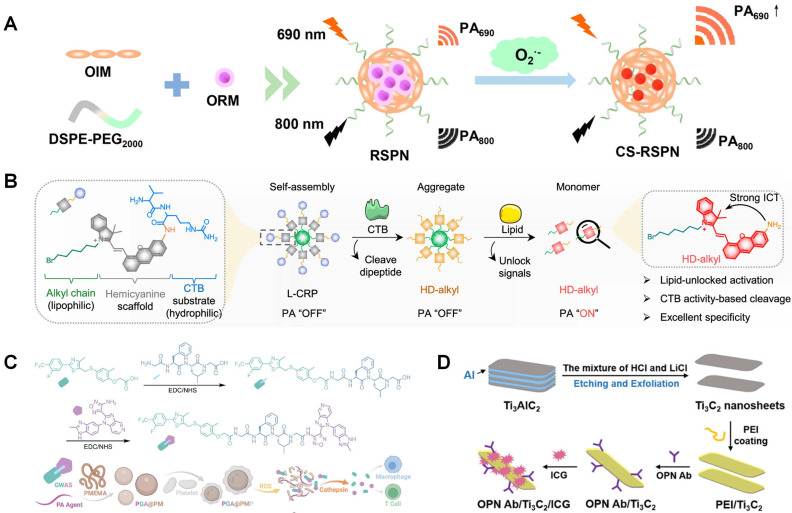
(**A**) One-step self-assembly of RSPN. (**B**) Chemical structure and the responsive progress of lipid-unlocked CTB responsive probe (L-CRP). (**C**) Synthesis route of GWAS and PGA@PMP, and its dual responsiveness to ROS and cathepsin B. (**D**) Schematic illustration of the preparation of OPN Ab/Ti_3_C_2_/ICG nanoprobes. (**A**) adapted with permission from 154 copyright 2021, American Chemical Society. (**B**) adapted with permission from 156 copyright 2023, American Chemical Society. (**C**) Adapted with permission from 157 copyright 2023, Wiley. (**D**) adapted with permission from 159 copyright 2020, Wiley.

Among these probes, semiconductive polymers, especially π-conjugated polymers, have delivered an impressive diagnostic outcome in malignant tumors and cardiovascular diseases with favorable accuracy and few background interference, which grants photoacoustic imaging a strong potential in clinical diagnostic practice. Ma *et al.* reported ratiometric semiconducting polymer nanoparticle (RSPN) for PAI of pneumonia induced aortic atherosclerosis 154. It is found this nanoparticle has an enhancement PAI signal at about 690 nm. As illustrated in Figure 6A, the optimized nanoparticle contains both O_2_^•-^-responsive molecule (ORM) and O_2_^•-^-insensitive semiconducting polymer molecule (OIM). ORM can react with O_2_^•-^ and turn on emission at 690 nm, which makes it be used as a probe to analyze oxidative stress in atherosclerotic plaque. Similarly, a π-conjugated polymer (PMeTPP-MBT) based on 3,6-bis(4-methylthiophen-2-yl)-2,5-bis(2-octyldodecyl) pyrrolo [3,4-c] pyrrole-1,4(2H,5H)-dione is designed as a novel photoacoustic contrast agent. Meanwhile, it is combined with SS-31 peptide and astaxanthin followed with oxidized dextran coating. This well-designed nanoparticle is studied as theranostic agent in Xu's research 155. The nanoparticle degradation can be triggered by low pH microenvironment and over expressed ROS. SS-31 peptide can inhibit ROS generation and restoration, when astaxanthin can increase the expression of ABCA1/G1 in foam cells.

As forementioned, active targeting is crucial in nanoprobe design and studied. Ma *et al.* reported cathepsin B (CTB) targeted PAI nanoprobe for atherosclerosis imaging (Figure 6B) 156. Multilayer nanoparticle is synthesized including self-assembled alkyl chain core, scaffold hemicyanine sublayer, and CTB substrate external surface. With the degradation of nanoparticles, PA response will be switched on as shown in Figure 6B. It existed as an aggregate before and after incubation with CTB in hydrophilic environments. L-CRP could be recognized by CTB and release HD-alkyl as a monomer in lipophilic environments, resulting in strong PA signals This CTB targeted nanoprobe can keep silence in lipid-deficient environments, such as M1 type macrophages and Lipopolysaccharide-induced inflammatory lesions. So, it can be used to measure CTB activity in lipophilic environments. Ma *et al.* reported platelet membrane coated PAI theranostic contrast. This nanomaterial can release immune-regulate complex GW0742-AS2863619 (GWAS) in the reactions with ROS and cathepsin B (Figure 6C), which is designed based on the regulation of GW0742 (GW) on macrophages as well as AS2863619 (AS) on T cells. It indicates that GWAS can regulate both M2 macrophages and T cells, simultaneously inhibiting the foam cell formation and rebuilding a well-balanced immune microenvironment 157. Gao *et al.* designed two types of near-infrared fluorescence probes loaded BSA nanoparticle for PAI, which can separately react with GSH and H_2_O_2_ and show absorbance at 765 and 680 nm. This dual responsive nanoparticle can further improve specificity and sensitivity 158. Ge *et al.* reported OPN antibody and ICG (NIR fluorescence molecules) co-assembled Ti_3_C_2_ nanosheets as PAI contrast for atherosclerosis (Figure 6D). With the help of OPN Ab, this nanoparticle can effectively target to foam cell and vulnerable plaque 159.

Compared with optical imaging, PAI can significantly improve the penetration depth. However, its penetration depth is still limited by the utilization of laser in a range from visible to NIR. It can be found some progress is done by applying active targeting nanoparticles in small animal model. To overcome the penetration problem, intravascular PAI can be a potential solution for coronary atherosclerosis. Nevertheless, noninvasive method may be much preferred compared with this invasive intravascular method. And the contrast agents with strong absorption in NIR-Ⅱ can also be an option to get deeper penetration for future development.

## 7. Multimodality Probes

The most popular image modalities in clinic contain CT, MRI, PET, Ultrasound and SPECT. Each single modality has its own unique pros and cons (Table 4). For instance, SPECT has excellent sensitivity and tissue permeability, but limited spatial resolution. And MRI can give images with high spatial resolution but relatively low sensitivity 160. The paradox of modality selection in clinical imaging process is that the modalities with high sensitivity have relatively low resolution, while those with high resolution usually provide poor sensitivity. Infusing multiple modalities has been one of the most attractive ideas to simultaneously realize high resolution and high sensitivity, and provide more accurate and reliable detection of disease lesions 161. The first fused PET/CT instrument was commercialized in 2001, which was developed in 1998 by Townsend's group. Around the same time, the fusion of PET and MRI was proposed. However, the first prototype of PET/MRI for human scale scanner was not available until 2007 for the great economic and engineering challenges. The main technical problem is caused by the application of magnetic field, which makes impossible to simultaneous acquiring both signals for PET and MRI 162. The successful commercialization of PET/CT and PET/MRI ignites the surging development of multimodality probe studies. Multimodal probes with two or more imaging agents can help to overcome the limitations and disadvantages of single modality and provide more details for atherosclerotic diagnosis.

### 7.1 MRI/CT nanoprobes

**Figure 7:**
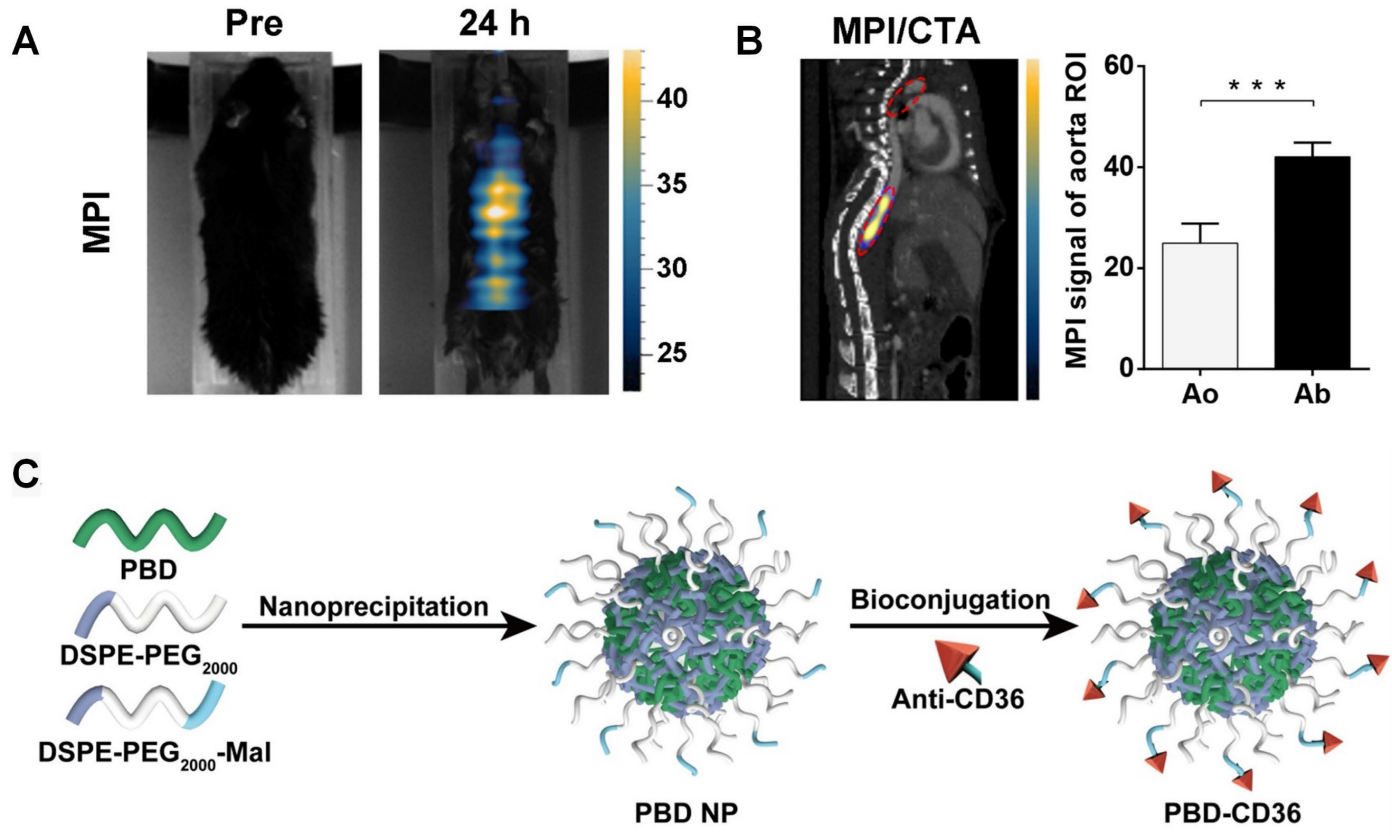
(**A**) MPI imaging of active MPO in aorta atheroma of ApoE^-/-^ mice before and 24 h post-injection of 5HFeC NPs; (**B**) Black ellipse indicates ROI in the aorta. MPI/CTA imaging of active MPO in the aorta of atherosclerotic mice and quantification of MPI signal in aortic arch and abdominal aorta. (**C**) Schematic illustration of the preparation of anti-CD36 decorated semiconducting polymer nanoparticles. (**A**) and (**B**) adapted with permission from 165 copyright 2021, Ivyspring International Publisher. (**C**) adapted with permission from 168 copyright 2020, Ivyspring International Publisher.

Ni *et al.* designed NaHoF_4_ nanoparticle for both CT and ultra-high field MRI imaging. Comparing with single CT imaging, the combination of CT and MRI can enhance the soft-tissue contrast. Meanwhile, the bone and calcification imaging capability is better than a single MRI 163. NaHoF_4_ nanoparticles with different sizes were studied from 3 nm to 29 nm. All these nanoparticles showed good CT and MRI imaging abilities. Curie mechanism has been demonstrated to make a main contribution to T2 MRI performance of NaHoF_4_ NPs <7 nm in diameter, while dipolar mechanism has been evidenced to be predominant as the size becoming larger. NaHoF_4_ exhibits a sharper signal enhancement compared with Iobitridol at different concentrations at 120 kVp 164. Tong *et al.* designed Fe_3_O_4_ nanoparticle for active myeloperoxidase detecting, which was a potential inflammatory marker of vulnerable plaque 165. 5-hydroxytryptamine was hired as an imaging biomarker which can self-oligomerize in inflamed tissues. In their research, this nanoparticle was used as multimodal imaging platform for MRI, and CT. In their research, magnetic particle imaging (MPI) is hired to evaluate the specificity and sensitivity of nanoparticle. In contrast to MRI, MPI allows simultaneous measurement of the harmonics of the magnetization change during particle excitation. Thus, MPI enables 3D image series in real time, enhanced spatial resolution and sensitivity. The MPI/computed tomography angiography (CTA) images in Figure 7A and B reveals the high concentration of Fe_3_O_4_ based nanoparticle in abdominal aorta. And the quantitative results also give an enhanced signal in abdominal aorta.

### 7.2 PAI/Ultrasound probes

As discussed, the application of PAI in clinic is hindered by the long-scan time and limited imaging depth (1-20 mm) 166. To shorten imaging time and deepen detecting distance, PAI is designed with transducer arrays with multiple elements. Combined with PAI, αvβ3-integrin-targeted ultrasmall gold nanorods (AuNRs) with cRGD show good detection ability to high-risk plaques. Liu *et al.* prepared a gold nanorod based nanoparticle for PAI/Ultrasound imaging. The cRGD labelled AuNRs result in an enhanced PA signal compared with PEG-gold nanoparticles from 2 h to 24 h 167. Xie *et al.* designed semiconducting polymer nanoparticles decorated with anti-CD36 (PBD-CD36) 168. It shows an absorption of 1064 nm, which can avoid the signal in biological tissues and afford good tissue penetration and a high signal-noise ratio. As shown in Figure 7C, this nanoparticle is synthesized via nanoprecipitation method. The mixed PBD and DSPE-PEG THF solution is injected into water and followed with sonication. PBD and DSPE-PEG precipitate and form nanoparticles due to their hydrophobic properties. The obtained nanoparticles are further conjugated with anti-CD36 via click reaction. Their results show enhanced PA signal in both left and right carotid arteries 24 h post injection.

### 7.3 SPECT/CT probes

Different with PET, SPECT method images the tissue and body samples based on collecting gamma rays emitted from radio isotopes. And it usually shows lower sensitivity than PET for the low ratio of photons that can transmit through collimators, which can reject the photons out of a small angular range. And to improve the spatial resolution, pinhole or multipinhole cameras are induced in SPECT system, which would further decrease the sensitivity leading to at least one order magnitude lower than PET sensitivity. And to provide an anatomical reference, CT or MRI are adopted in multimodality system. This combined SPECT/CT facilitates anatomical localization of the radiopharmaceutical to differentiate physiological uptake from that associated with disease and patient-specific attenuation correction to improve the visual quality and quantitative accuracy of the SPECT image 169, 170.

As discussed above, gold nanoparticle has been viewed as an ideal CT contrast. However, a relatively large amount of gold nanoparticle is required for CT limited low sensitivity. The combination of CT and SPECT makes it can not only accurately detect the location of plaque, also monitor the pathological changes via SPECT intensity changes. ^99m^Tc decorated gold nanoparticle is used as SPECT/CT contrast in Li's study 171. This nanoparticle material is also labelled with Annexin V for apoptotic macrophage targeting. The relative ratios of normal and apoptotic macrophages can be an indicator to quantify the pathological process.

**Figure 8:**
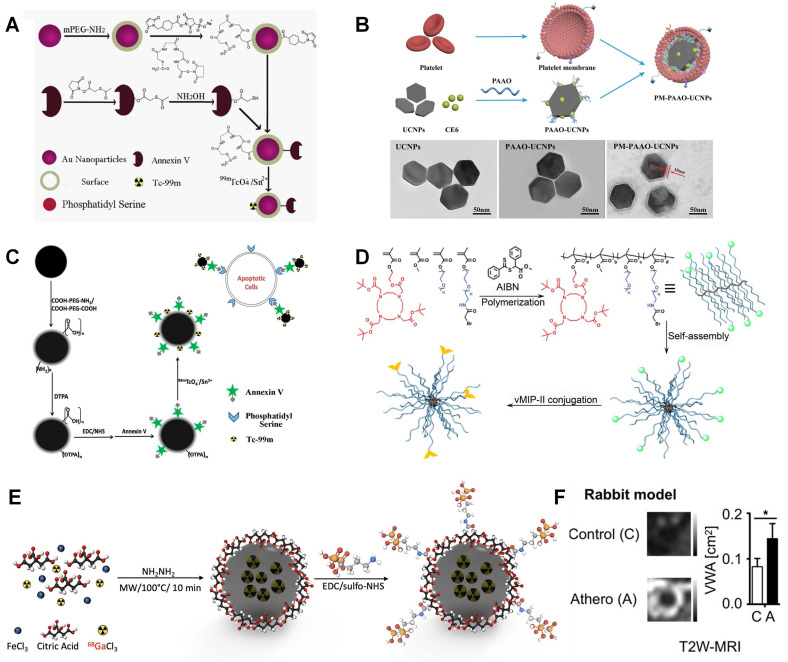
(**A**) Synthetic procedure of ^99m^Tc-GNPs-Annexin V. (**B**) Schematic illustration showing the composition of PM-PAAO-UCNPs, and TEM images of UCNPs, UCNPs loaded in micelles (PAAO-UCNPs), and platelet member-coated PAAO-UCNPs (PM-PAAO-UCNPs. (**C**) Schematic presentation of the procedures for preparing ^99m^Tc-DTPA-USPIO-Annexin V. (**D**) Illustration of vMIP-II-comb (α-bromide, vMIP-II) synthesis. (**E**) Microwave two-step synthesis of ^68^Ga-IONP-Alendronate. (**F**) Vessel wall area in atherosclerotic rabbits and control animals, as was vascular permeability measured by 3D dynamic contrast enhanced-magnetic resonance imaging (3D DCE-MRI). **(A)** adapted with permission from 171, copyright 2016 Elsevier. **(B)** adapted with permission from 172, copyright 2021 Wiley. **(C)** adapted with permission from 102, copyright 2015 American Chemical Society. (**D**) adapted with permission from 174 copyright 2016, Society of Nuclear Medicine and Molecular Imaging. (**E**) adapted with permission from 176 copyright 2021, American Chemical Society. (**F**) adapted with permission from 75, copyright 2019 American Chemical Society.

As exhibited in Figure 8A, the obtained gold nanoparticles are fully covered with PEG-NH_2_ and then modified with two linkers. MAG3 is used for Annexin V linking via the reaction between NHS and NH_2_ groups, while sulfosuccinimidyl 4-(N-maleimidomethyl)cyclohexane-1-carboxylate is used to form coordination with ^99m^Tc. This Annexin V labelled nanoparticle can effectively target to apoptotic cells rather than macrophages, which can help to monitor pathological process. Ma *et al.* designed lanthanide doped upconversion nanoparticles (UCNPs), which is entrapped by platelet membrane. Lanthanide doped UCNPs and chlorin e6 are entrapped by polyacrylic acid-n-octylamine (PAAO) micelles (Figure 8B). The coating platelet membrane is verified by TEM imaging 172. Similarly, Liu *et al.* designed anti-CD11b and ^99m^Tc labelled MAG3 nanoparticle for SPECT/CT imaging. A high sensitivity is achieved in aortic arch and renal artery plaque in SPECT/CT 173.

### 7.4 SPECT/MRI probes

Similar with the combination of SPECT and CT, SPECT/MRI can also realize both high spatial resolution and high sensitivity. Cheng *et al.* designed ^99m^Tc labelled Fe_3_O_4_ nanoparticle and utilized in SPECT/MRI imaging to target apoptotic macrophages 102. As shown in Figure 8C, Fe_3_O_4_ nanoparticles are coated with PEG and followed with DTPA and NHS modification for ^99m^Tc and Annexin V conjugation, separately. This well-designed nanoparticle system can specifically target vulnerable plaques containing apoptotic macrophages.

### 7.5 PET/CT probes

Bala *et al.* developed a PET/CT tracer to target VCAM-1, which is an important biomarker on endothelial cells. Anti-VCAM-1 nanobody (cAbVCAM-1-5) was radiolabeled with ^18^F. It makes this tracer can reflect inflammation progress 68. Luehmann *et al.* designed ^64^Cu labelled viral macrophage inflammatory protein Ⅱ based nanoparticle for PET/CT imaging 174. This comb like nanoparticle is a good example to assess plaque progression by utilizing specific detection of chemokine receptors. As shown in Figure 8D, vMIP-Ⅱ is conjugated to a poly(methyl methacrylate)-core/PEG-shell amphiphilic nanoparticle through controlled conjugation and radiolabeled with ^64^Cu for PET imaging. The quantified PET/CT image results exhibit good targeting benefits. And the signal can be significantly decreased by blocking the receptors in ApoE^-/-^ mice, which strongly support this vMIP-Ⅱ related pathology.

### 7.6 PET/MRI probes

The integration of PET and MRI brings together the advantages of each individual modality. The outstanding soft tissue contrast and 3D imaging capability of MRI can be a sufficient addition to PET. Three nanobody radiotracers were studied that accumulate in atherosclerotic plaque including VCAM-1, lectin-like oxidized low-density lipoprotein receptor (LOX)-1, and macrophage mannose receptor. Senders *et al.* found that ^64^Cu labelled macrophage mannose receptor nanobody showed highest radioactivity concentration in aorta-to-blood ratio 175. Garcia *et al.* designed ^68^Ga-CM246 targeting fibrin cells for PET/MRI imaging. Both tissue-to-background (back muscle) ratios and SUVs were significantly higher in the thrombotic versus nonthrombotic group 77. Pellico *et al.* designed bisphosphonate-based nanoparticles with ^68^Ga doped iron nanocore to characterize microcalcifications in atherosclerotic lesions 176. As illustrated in Figure 8E, ^68^Ga labeled iron oxide particle is synthesized via microwave assisted synthesis method. Then, the bisphosphonate moiety (alendronate sodium) is coupled with nanoparticle via sulfo-NHS chemistry. It shows good targeting effect to most common calcium scales present in microcalcifications, especially to hydroxyapatite, which is a main compound in atherosclerosis microcalcifications. Compared with citrate labelled material, the nanoparticle with alendronate gives much better affinity to hydroxyapatite. Lobatto *et al.* synthesized ^89^Zr-labelled liposomes for atherosclerotic vessel wall imaging 75. Both PET/CT and PET/MRI were carried out for imaging and quantifying. A significant accumulation is observed in atherosclerotic lesion. Both T2W-MRI and 3D-DCE-MRI give enhanced signal compared with control group (Figure 8F). And a long retention time can also be monitored for 15 days due to the long half-life time of ^89^Zr.

### 7.7 MRI/ Fluorescence probes

Zhang *et al.* synthesized ASA6 antibody conjugated NaNdF_4_@NaGdF_4_ nanoparticles for both MRI and the second region near infrared (NIR-Ⅱ, 1,000-1,700 nm) fluorescence imaging 123. ASA6, a scFv antibody, can bind to oxidized LDL and atherosclerotic plaque. NaNdF_4_@NaGdF_4_ nanoparticle responses to NIR excitation at 808 nm and gives an emission at 1340 nm. The fluorescence imaging in NIR-Ⅱ window (1000-1700 nm) shows much better permeation than traditional near infrared wavelength (NIR-I, 650-950 nm). NIR imaging is preferred in research for its fast imaging, nonionizing radiation, and low cost. But it is still a great challenge for human coronary imaging for the limited penetration in human tissues. And MRI integration can effectively enhance the penetration for its good tissue penetrating in human body imaging.

### 7.8 CT/Fluorescence probes

Chen *et al.* reported Bi_2_S_3_ nanoparticle, CdSe and ZnS quantum dots for both CT and fluorescence imaging. All these nanomaterials are coated with 1,2-Dipalmitoyl-sn-glycero-3-phosphoethanolamine-N-[methoxy(polyethylene glycol)-2000. The short emission wavelength around 650 nm makes it a limited optical imaging capability. Images from *in vivo* CT testing are obtained from 2 h to 24 h indicating a long retention time for these materials 177.

Historically, anatomical structure imaging and functional process research developed in a path distinct from each other. Although combining different modalities can significantly improve diagnostic accuracy and reveal disease pathology, it raises great challenges in both software and hardware fusion 178. The successful commercialization of combined devices like PET/CT, PET/MRI triggers numerous studies about multimodal probes for various diseases. To simultaneously achieve high resolution and sensitivity, radioactivity imaging methods, like SPECT and PET, are preferred to combine with methods providing good spatial resolutions, especially CT and MRI (Table 5). Due to the high sensitivity of radioactive imaging methods, only tiny amounts of radioactive isotopes are used in nanoparticle design and synthesis. It is necessary to add different chelators depending on radioactive isotopes. It is another option to mix isotopes with contrast materials for CT and MRI. Namely, designing radioactive isotopes doped materials as multimodal probes. A good specificity is required in the design due to the utilization of radioactive isotopes, which cause radiation risks and high costs. Optical imaging or fluorescence imaging is another choice for its high sensitivity and fast imaging process. Its spatial resolution can be greatly enhanced by combining with CT or MRI. It is a favor for laboratory research, especially for small animal experiments. However, the application in clinic is highly inhibited by its limited penetration ability. It is no doubt that multimodality imaging will play a leading role in clinical applications of diagnostic imaging research.

## 8. Challenges and Perspectives

As discussed, many molecular imaging nanoprobes are reported in recent years and show great potentials for clinical applications. However, some challenges are still existing.

Regarding nanoprobe design, 1) these nanomaterials are usually functionalized with single ligand for one-to-one targeting strategies, which heavily relies on the binding affinity of individual ligands. Due to the unique properties of atherosclerosis, off-target effects may be very high. For example, ^18^F-FDG can be easily affected by hypoxia and increased myocardial muscle activity. The high-level glucose uptake in heart and brain can also decrease the amount of signal intensity in plaques. Multiple targeting nanoprobe can be a potential candidate to improve accumulation efficiency and decrease off-target effects.

2) Most studies focus on the targeting to macrophages. However, macrophage is widely distributed in body, which would increase off-targeting effects. And some other cells are also confirmed participating in the development of atherosclerosis like endothelium, platelet, monocyte, foam cell and so on. And more efforts can be devoted on relevant targets such as VCAM-1, profilin-1, CD44 receptor, αⅡbβ 3 integrin, ανβ3 integrin and CCR2. The endothelial cell dysfunction and leukocytes migration happen at the early stages of atherosclerosis development. And it is important to develop effective nanoprobes for diagnosis and treatment at these stages.

3) Good stability and fast degradation are conflicting to each other. Long retention time in blood can greatly contribute to high accumulation in plaque and high utilization of nanoprobes, especially for radioactive isotopes. However, the longtime radiation exposure may cause potential hazards. The good stability of gold nanoparticles makes it a good CT contrast. But it may also cause safety concerns about long time accumulation and toxicity. And the residual contrasts in body may also lead to interference for following examinations, especially for atherosclerosis.

Some challenges cannot be ignored in animal research. 1) Toxicity evaluation is often insufficient in relevant research, while it is crucial in clinical translations. It can be found that *in vivo* toxicity is usually tested after one injection in relevant studies. It is not sufficient for long term safety and toxicity evaluation. Generally, three levels safety issues should be considered including local overexposure, unexpected nanomedicine-related toxicity in humans, and nanomaterial related immunological responses 190-192.

2) The understanding about atherosclerosis can also inhibit the application of nanoparticles in molecular imaging. To identify a more valuable hallmark in molecular level not on structural imaging (vulnerable plaque) for clinical decision making can be a guidance for nanoparticle design. And a clear standard for atherosclerosis quantification or analysis can also help and promote the early-stage monitoring for atherosclerosis developments.

3) Vulnerable plaque indicates a plaque with an unpredictable rupture risk causing adverse cardiovascular events such as acute myocardial syndrome and even sudden cardiac death. And there is currently no definitive proof to prospectively identify vulnerable plaques. Furthermore, animal models are also lack of spontaneous plaque rupture. More efforts are required to analyze the complicated physical and biochemical microenvironments of vulnerable plaque. Researchers also should focus on the studies of the morphological features and composition of the plaques to evaluate the vulnerability and potential risk for rupture by histological approaches 193.

As for commercialization and clinical trials, the successful scale-up and manufacturing of a nanocomplex present unique challenges comparing with conventional small molecule drugs. 1) The synthesis of nanoparticles at the lab or small scale usually involves organic solvents, sonication, emulsification, crosslinking and so on. It will be useful to consider what methods can be feasible to be scaled up. It can be noticed that click chemistry strategies are widely applied in discussed reports. This may be a hint for reaction design to synthesize multitargeting nanoprobes.

2) High reproducibility should also be taken into consideration when the researchers design synthesis procedure. Continuous efforts are required to simplify synthesis procedures and promote commercialization. As a continuous synthesis method, microfluid manufacture displays great advantages for complex synthesis, like good controllability and reproducibility. Microfluidic devices usually feature continuous flow, precise control of reaction time, high-temperature controllability, and shorter diffusion distance in a microchannel, which are advantageous to produce microparticles and nanoparticles 194, 195.

3) It can be noted that peptide and antibodies are widely used in these studies. These materials required strict conditions for storage and unique reaction conditions. Or these materials will lose their biological activities. Looking for different targeting molecules is also important to overcome this disadvantage.

4) Nanoparticle has three-dimensional structure in nanometer scale. It is important to identify key parameters to standardize synthesis procedure and quality control. For example, the exposure of targeting ligands on nanoparticle surface is crucial for achieving effective targeting. So, it is necessary to develop a method quantifying exposed ligands and ensuring a good reproducibility.

## 9. Conclusions

Atherosclerosis is a common cardiovascular disease that slowly progresses throughout life. The atherosclerotic plaque growth may obstruct blood and lead to heart attack or stroke. Early detection and intervention are important for atherosclerosis treatments. With the fast development of molecular imaging methods, it becomes possible to detect and image atherosclerotic plaques, especially vulnerable plaques in progressing. Several molecular imaging methods are briefly reviewed in this manuscript including CT, PET, MRI and so on. The application of targeting probes is of importance in improving image quality in molecular imaging studies. It can be found relevant studies mainly focus on active targeted imaging for the unique properties of atherosclerosis. Vascular smooth muscle cell, endothelial cell and macrophage are major targeted sites in the design of active targeted nanomaterials. And various targeting probes are studied, such as antibody, peptide, genes, cell membranes. The nanoprobes are usually specific for individual modality. For instance, iodine and gold nanoparticles have gotten numerous studies as CT contrast. Gd and Fe based compounds are viewed as the most promising candidates for MRI characterization. ^18^F related complexes are widely utilized in clinic for its good uptake and relatively short half-life time. ^99m^Tc is the most hired isotope for SPECT imaging. Exploring and extending the ranges of contrasts is another way to perfect the molecular imaging probe design and development. And theranostic nanomaterial is another direction for the development of atherosclerotic related nanomaterials.

Combining different modalities has been one of the most powerful strategies to enhance diagnostic accuracy and reveal disease pathology, especially for cardiovascular diseases. Several fused imaging equipment have been available in clinic, such as PET/CT and PET/MRI. It also requires designing and developing new nanoprobes for fused modalities. The goal is to simultaneously enhance the sensitivity and resolution. Generally, radiation related methods are chosen as the high sensitivity modality, while CT and MRI are used to improve imaging resolution. This proposes new requirements and challenges for nano probe design. Polymer matrix is usually preferred to conject different tracers. And the utilization of unique chelators is important for radioactive isotopes due to the ionic formation and tiny amount.

It is no doubt to say that the development and application of molecular imaging technology have greatly contributed to the diagnosis and treatment of cardiovascular diseases, especially atherosclerosis. However, some challenges still exist inhibiting the clinical transition and commercialization. We hope this review can contribute to the studies about atherosclerosis and inspire more excellent studies.

## Figures and Tables

**Table 1:** CT contrasts for atherosclerosis

Tracer	Matrix	Targeting ligands	Targeted site	Ref.
Gold	Glycol-chitosan	Fibrin-targeting peptides	Fibrin cells	41
Gold	PEG/PCCP	-	-	31
Gold/Pd	PEG	Folic acid	Folate receptor	33
Gold	11-MUA/16-MHA/MPEG/PCOOH/PNH_2_	-	Macrophage	28
Gold	-	-	-	42
Gold/Silver	BSA	-	-	32
Iodixanol	1,2-dipalmitoyl-sn-glycero-3- phosphocholine, cholesterol, 1,2-distearoyl-sn-glycero-3-phospho-ethanolamine-N-[methoxy (polyethylene glycol)-2000]	-	-	24
Iodixanol	DPPC/Cholesterol	PS	Macrophages	25
BaGdF_5_	Metal phenolic network	-	-	43
Exitron nano 12000	-	-	-	44

**Table 2:** PET and PET/CT contrasts for atherosclerosis

Tracer	Matrix	Targeting ligands	Targeted site	Ref.
^18^F	FDG	Cytotoxic T-Lymphocyte Associated protein	T cells	65
^18^F	FDG	-	Vulnerable lesions	66
^18^F	NaF	-	Calcium deposition	67
^18^F	-	Anti-VCAM-1 nanobody	VCAM-1	68
^18^F	FDG	-	-	69
^18^F	PEGylated mesoporous Silica	-	Macrophage	70
^18^F	FDG	-	Abdominal aorta	48
^18^F	Mannan	-	Mannose receptor	71
^64^Cu	PEG	C-atrial natriuretic factor	Natriuretic peptide clearance receptor	72
^64^Cu	Gold	Extracellular loop 1 inverso peptide	CCR2	73
^64^Cu	ATSM	-	Hypoxia/ low-oxygen-tension cells	57
^64^Cu	Polyglucose	-	Cardiac/Arterial/Pulmonary macrophages	74
^89^Zr	Chitosan nanoparticles	-	Leukocytes	64
^89^Zr	PEG/Phospholipid	-	Atherosclerotic vessel wall	75
^68^Ga	2-(p-isothiocyanatobenzyl)-NOTA	Mannose	Mannose receptor-positive inflammatory cells	76
^68^Ga	CM246	-	Carotid plaque	77
^124^I/^125^I	Gold	-	-	78

**Table 3:** MRI contrasts for atherosclerosis

Tracer	Matrix	Targeting ligands	Targeted site	Ref.
Gd	-	Glucagon-like peptide-1 receptor agonists	Smooth muscle cell	97
Gd	Liposome	THI0567	α4β1 integrin	98
Gd	High density lipoprotein	Synthetic apolipoprotein A-I peptides	Macrophages	99
Gd	DSPE-PEG	CREKA	Clot	84
Gd	Hybrid lipid-latex	PS and cholesterol-9-carboxynonanoate	Macrophages	100
Gd	Amide-forming potassium acyltrifluoroborate	Apolipoprotein-mimetic peptide	Atherosclerotic plaques	83
Gd	Glutathione	PP1	SR-AI receptor	82
Gd	DTPA	APT_FN-EDB_	Extra domain B of fibronectin	101
Fe_3_O_4_	-	Macrophage membrane	α4β1 integrin VCAM-1	87
Fe_3_O_4_	PEG	Annexin V	Apoptotic macrophages	102
Fe_3_O_4_	PEG	Mouse anti-rabbit IL-6 monoclonal antibody and a nonspecific IgG antibody	Foamy macrophages	103
Fe_3_O_4_	PEG	IgG antibodies and anti-CTGF polyclonal	Macrophages	91
Fe_3_O_4_	PEG	scFv-Fc antibody	Galectin 3	89
Fe_3_O_4_	DSPE-PEG3400-Maleimide	scFv-Fc TEG4-2C antibody	Atheroma plaque	104
Fe_3_O_4_	PEG-PEI	Profilin-1 antibody	Vascular smooth muscle cell	90
Fe_3_O_4_	DSPE-PEG(2000)-maleimide	Fbrin binding peptide	Thrombosis	105
Fe_3_O_4_	Meso-2,3-Dimercaptosuccinic acid	Polyclonal profilin-1 antibody	Profilin-1	106
Fe_3_O_4_	Dextran	MCP-1 peptides	CCR2	107
Fe_3_O_4_	DMSA	Osteopontin antibody	Foamy macrophages	108
Fe_3_O_4_	PLGA/perfluorohexane	Dextran sulfate	SR-A	109
Fe_3_O_4_	SiO_2_	VHPKQHR peptide	Endothelial cells	110
Iron oxide	-	HA	CD44-expressing cells	94
Iron oxide	SiO_2_	PP1 peptide	Fomay macrophage	111
Iron oxide	PEG	Dingle-chain antibodies (scFv)	GPⅡb/Ⅲa receptors	112
Iron oxide	PEG	Human IgG4 antibody, rIgG4 TEG4	Human activated platelets	113
Iron oxide	PEG	DNA	Macrophages	114
Iron oxide	poly-l-lysine	endothelial progenitor cell	intima of vascular lesions	115
Iron oxide	PLGA/DSPE-PEG	Stearyl-TPP and stearyl-mannose	Macrophages	116
Iron oxide	PEG and dextran	scFv antibody fragments	Activated platelets	117
Iron oxide	Pluronic	cRGD peptide	αvβ3 integrin	88
Iron oxide	Pluronic	Collagen IV targeting peptide	Collagen type IV	88
Fe(Ⅲ)	Tannic acid	Dopamine-modified hyaluronic acid (HD)	CD44-expressing cells	118
Gd/Eu	-	Short peptide	CCR2	119
Superparamagnetic iron oxide	Carboxymethyl dextran	Fucoidan	Endothelial cells and platelets	120
Superparamagnetic iron oxide	Dextran	-	-	121
Superparamagnetic iron oxide	poly(maleicanhydride-alt-1-octadecene) (PMAO)/ poly(ethyleneimine) (PEI)/ alendronate	-	-	122
Gold-coated iron oxide (Fe_3_O_4_)	-	Anti-CD163 antibody	CD163	85
CeO_2_ and Fe_3_O_4_	Chitosan, Poly (acrylic acid)	-	-	93
TiO_2_	EDC-NHS	MCP-1 antibody	Monocyte and endothelial	95
MnFe_2_O_4_	PLGA	Anti-VEGFR-2 antibody	Vascular endothelial growth	96
NaNdF_4_@NaGdF_4_	Phosphorylated brush polymer	scFv antibody, ASA6	Oxidized LDL/ Oxidation-specific epitopes	123
NaGdF_4_:Yb, Er	PEG	Anti-MARCO antibody	Macrophage receptor with collagenous receptor	124
-	PLGA	Platelet membrane	Atherosclerotic plaques	125

**Table 4:** Advantages and limitations of atherosclerosis imaging techniques.

Modality	Advantages	Limitations	Refs.
PET/SPECT	High biologic sensitivityHigh reproducibility over the short term	Radiation exposure Low spatial resolution Expensive	46,47
MRI	High soft-tissue contrast High resolution Suitable for serial studiesNo ionizing radiation	Multi-parametric imaging sequences Long scan timeNot suitable for patients with metal implanted devices	79
CT	Relatively high resolution Minimally invasive High specificity High reproducibility	Low sensitivity Radiation exposure	20
Ultrasound	Low cost No radiation High speed Portable Accessible Minimally invasive	Acoustic shadowing Post-imaging analysis Artifacts	126,128
Fluorescence	Relatively high resolution Low cost No radiation Information on lipid content of plaque	Limited imaging depth Self-fluorescence	50,53,106

**Table 5:** Multimodality contrasts for atherosclerosis

Modality	Tracer	Matrix	Targeting ligands	Targeted site	Ref.
MRI/CT	NaHoF_4_	PEG	-	-	164
	Fe_3_O_4_	PEG	5-hydroxytryptamine	myeloperoxidase	165
PAI/Ultrasound	Gold	PEG	cRGD	αvβ3 integrin	167
	PBD	PEG-DSPE	CD36	Atherosclerotic plaque	168
CT/Fluorescence	Bi_2_S_3_	PEG-DSPE	-	-	177
MRI/Fluorescence	Fe_3_O_4_/ Rhodamine	PEG	-	Aortic plaque	179
	Gd/Nd	-	scFv antibody	oxidized LDL	123
	Iron oxide/Rhodamine B	PEG	-	-	180
PET/CT	^64^Cu	PMMA/PEG	vMIP-Ⅱ	Macrophage	174
	^18^F	Nanobody	VCAM-1	Atherosclerotic plaque	68
PET/MRI	^89^Zr	High-density lipoprotein	CD40-TRAF6i	TRAF6	181
	^68^Ga	CM246	fibrin-specific peptide	Fibrin	77
	^64^Cu/^68^Ga	Nanobody	VCAM-1	Atherosclerotic plaque	175
	^64^Cu/^68^Ga	Nanobody	LOX-1	Atherosclerotic plaque	175
	^64^Cu/^68^Ga	Nanobody	Mannose receptor	Atherosclerotic plaque	175
	^89^Zr	Liposome	PEG/Phospholipid	-	75
	^89^Zr	HA	-	Atherosclerotic aortas	182
	Fe/^68^Ga	Hydroxyapatite/ Citric acid	-	Microcalcifications	176
	Fe_3_O_4_/^68^Ga	2, 3-dimercaptosuccinnic acid/ NOTA	GEBP11	Neovascularization of endothelial cells	183
SPECT/MRI	Iron oxide/^99m^Tc	PEG	Annexin V	apoptotic macrophages	102
SPECT/CT	^99m^Tc	MAG3	Anti-CD11b antibody	CD11b antibody	184
	^99m^Tc	PEG	Annexin V	Apoptotic macrophages	171
	^125^I/^131^I	PEG	Folic acid	Folate receptor	33
	Ce6	PAAO	Platelet membrane	Foam cells	172
	^99m^Tc	-	Duramycin	Apoptotic macrophages	185
	^99m^Tc	MAG3	H-ferritin	Macrophage	187
	^111^In	DOTA	JR11	Somatostatin Subtype Receptor 2	186
	^111^In	dextran	D-mannose	Mannose receptor	188
	^99m^Tc	MAG3	bevacizumab	VEGF-A	189

## Abbreviations

11-MUDA: 11-mercaptoundecanoic acid
16-MHDA: 16-mecaptohexadecanoic acid
^18^F-FDG: ^18^F-fluorodeoxyglucose
^18^F-NaF: ^18^F-fluoride
4-MB: 4-mercapto-1-butanol
ATSM: Diacetyl-bis (N^4^-methylthiosemicarbazone)
AuNRs: Gold nanorods
BSA: Bovine serum albumin
CCR2: C-C chemokine receptor type 2
CEUS: Contrast enhanced ultrasound
CREKA: Cysteine-arginine-glutamic acid-lysine-alanine
CT: Computed tomography
CTA: computed tomography angiography
CTB: Cathepsin B
CTGF: Connective tissue growth factor
DMPC: 1, 2-Dimyristoyls-sn-glycerol-3-phosphocholine
DOTAP: 1,2-Dioleoyl-3-trimethylammonium-propane
DPPC: Dipalmitoyl phosphatidylcholine
DSPE-PEG: 1,2-distearoyl-sn-glycero-3-phosphoethanolamine-N-methoxy(polyethyleneglycol)
DTPA: Diethylenetriamine-pentaacetic acid
GSH: Glutathione
EDC: 1-ethyl-3-(3-dimethylaminopropyl)carbodiimide hydrochloride
GWAS: GW0742-AS2863619
HA: Hyaluronan
HA-NPs: HA nanoparticles
ICP-OES: Inductively coupled plasma optical emission spectroscopy
IFN-γ: Interferon-γ
IMT: Intima-media thickness
IONPs: Iron oxide nanoparticles
IVUS: Invasive spectrum-intravascular ultrasound
LDL: low-density lipoprotein
Mal-PEG-NHS: Poly(ethylene glycol) (N-hydroxysuccinimide 5-pentanoate) ether N′-(3-maleimidopropionyl)aminoethane
MCP-1: Chemoattractant protein-1
M-CSF: Macrophage colony-stimulating factors
MPEG: Poly(ethylene-glycol) with methoxy
MPI: Magnetic particle imaging
MRI: Magnetic resonance imaging
MSA: Neomannosylated human serum albumin
MTEG: 11-mercaptoundecyl-tetra(ethylene glycol)
NIR: Region near infrared
OPN: Osteopontin
OIM: O2•--insensitive semiconducting polymer molecule
ORM: O2•--responsive molecule
PAAO: Polyacrylic acid-n-octylamine
PAI: Photoacoustic imaging
PCCP: Poly di(carboxylatophenoxy)phosphazene
PCOOH: Carboxylic acid
PEG-PLL: Polyethylene glycol-polylysine
PEI: Poly(ethyleneimine)
PET: Positron emission tomography
PFOB: Perfluorooctyl bromide
PLGA: Poly(lactic-co-glycolic acid)
PS: Phosphatidylserine
RGD: Arg-Gly-Asp
ROS: Reactive oxygen species
RSPN: Ratiometric semiconducting polymer nanoparticle
SCN-NOTA: 2-(p-isothiocyanatobenzyl)-1,4,7-triazacyclononane-1,4,7-triacetic acid
SPAAC: Strain-promoted alkyne azide cycloaddition
SPECT: Single-photon emission computed tomography
SRs: Scavenger receptors
SUVs: Standardized uptake values
TEM: Transmission electron microscopy
TNF: Tumor necrosis factor
TPP: Tripolyphosphate
UCNPs: Upconversion nanoparticles
VCAM-1: Vascular cell adhesion molecule-1
VWF: Von Willebrand factor

## References

[1] Mushenkova NV, Summerhill VI, Zhang D, Romanenko EB, Grechko AV, Orekhov AN (2020). Current advances in the diagnostic imaging of atherosclerosis: insights into the pathophysiology of vulnerable plaque. Int J Mol Sci.

[2] Libby P (2021). The changing landscape of atherosclerosis. Nature.

[3] Tanwi V (2022). Soundharya K. Eoin B. Decoding microRNA drivers in atherosclerosis. Biosci Rep.

[4] Björkegren JL, Lusis AJ (2022). Atherosclerosis: recent developments. Cell.

[5] Jebari-Benslaiman S, Galicia-García U, Larrea-Sebal A, Olaetxea JR, Alloza I, Vandenbroeck K (2022). Pathophysiology of atherosclerosis. Int J Mol Sci.

[6] Zhang X, Centurion F, Misra A, Patel S, Gu Z (2023). Molecularly targeted nanomedicine enabled by inorganic nanoparticles for atherosclerosis diagnosis and treatment. Adv Drug Delivery Rev.

[7] Anwaier G, Chen C, Cao Y, Qi R (2017). A review of molecular imaging of atherosclerosis and the potential application of dendrimer in imaging of plaque. Int J Nanomed.

[8] Prilepskii AY, Serov NS, Kladko DV, Vinogradov VV (2020). Nanoparticle-based approaches towards the treatment of atherosclerosis. Pharmaceutics.

[9] Dai T, He W, Yao C, Ma X, Ren W, Mai Y (2020). Applications of inorganic nanoparticles in the diagnosis and therapy of atherosclerosis. Biomater Sci.

[10] Chen L, Jiang Z, Akakuru O, Yang L, Li J, Ma S (2020). Recent progress in the detection and treatment of atherosclerosis by nanoparticles. Mater Today Chem.

[11] Kavitha M, Vallakeerthi N, Ramesh P, Reddy PM (2020). Role of metallic nanoparticles in cardiovascular disease. J Cardiovasc Dis Res.

[12] Younis NK, Ghoubaira JA, Bassil EP, Tantawi HN, Eid AH (2021). Metal-based nanoparticles: promising tools for the management of cardiovascular diseases. Nanomedicine.

[13] Lewis DR, Petersen LK, York AW, Zablocki KR, Joseph LB, Kholodovych V (2015). Sugar-based amphiphilic nanoparticles arrest atherosclerosis in vivo. Proc Natl Acad Sci U S A.

[14] Iverson NM, Plourde NM, Sparks SM, Wang J, Patel EN, Shah PS (2011). Dual use of amphiphilic macromolecules as cholesterol efflux triggers and inhibitors of macrophage athero-inflammation. Biomaterials.

[15] Mlinar LB, Chung EJ, Wonder EA, Tirrell M (2014). Active targeting of early and mid-stage atherosclerotic plaques using self-assembled peptide amphiphile micelles. Biomaterials.

[16] Luo Y, Guo Y, Wang H, Yu M, Hong K, Li D (2021). Phospholipid nanoparticles: therapeutic potentials against atherosclerosis via reducing cholesterol crystals and inhibiting inflammation. EBioMedicine.

[17] Chen J, Zhang X, Millican R, Creutzmann JE, Martin S, Jun H-W (2020). High density lipoprotein mimicking nanoparticles for atherosclerosis. Nano Convergence.

[18] Zakirov FH, Zhang D, Grechko AV, Wu WK, Poznyak AV, Orekhov AN (2020). Lipid-based gene delivery to macrophage mitochondria for atherosclerosis therapy. Pharmacol Res Perspect.

[19] Weissleder R, Mahmood U (2001). Molecular imaging. Radiology.

[20] Danad I, Fayad ZA, Willemink MJ, Min JK (2015). New applications of cardiac computed tomography: dual-energy, spectral, and molecular CT imaging. JACC Cardiovasc Imaging.

[21] He W, Ai K, Lu L (2015). Nanoparticulate X-ray CT contrast agents. Sci China Chem.

[22] Schöckel L, Jost G, Seidensticker P, Lengsfeld P, Palkowitsch P, Pietsch H (2020). Developments in X-ray contrast media and the potential impact on computed tomography. Invest Radiol.

[23] Barrett BJ, Katzberg RW, Thomsen HS, Chen N, Sahani D, Soulez G (2006). Contrast-induced nephropathy in patients with chronic kidney disease undergoing computed tomography: a double-blind comparison of iodixanol and iopamidol. Invest Radiol.

[24] Ghaghada KB, Ren P, Devkota L, Starosolski Z, Zhang C, Vela D (2021). Early detection of aortic degeneration in a mouse model of sporadic aortic aneurysm and dissection using nanoparticle contrast-enhanced computed tomography. Arterioscler Thromb Vasc Biol.

[25] Kee P, Bagalkot V, Johnson E, Danila D (2015). Noninvasive detection of macrophages in atheroma using a radiocontrast-loaded phosphatidylserine-containing liposomal contrast agent for computed tomography. Mol Imaging Biol.

[26] Donahue ND, Acar H, Wilhelm S (2019). Concepts of nanoparticle cellular uptake, intracellular trafficking, and kinetics in nanomedicine. Adv Drug Delivery Rev.

[27] Huang X, Peng X, Wang Y, Wang Y, Shin DM, El-Sayed MA (2010). A reexamination of active and passive tumor targeting by using rod-shaped gold nanocrystals and covalently conjugated peptide ligands. ACS Nano.

[28] Chhour P, Kim J, Benardo B, Tovar A, Mian S, Litt HI (2017). Effect of gold nanoparticle size and coating on labeling monocytes for CT tracking. Bioconjugate Chem.

[29] Chhour P, Naha PC, O'Neill SM, Litt HI, Reilly MP, Ferrari VA (2016). Labeling monocytes with gold nanoparticles to track their recruitment in atherosclerosis with computed tomography. Biomaterials.

[30] Choi HS, Liu W, Misra P, Tanaka E, Zimmer JP, Ipe BI (2007). Renal clearance of nanoparticles. Nat Biotechnol.

[31] Cheheltani R, Ezzibdeh RM, Chhour P, Pulaparthi K, Kim J, Jurcova M (2016). Tunable, biodegradable gold nanoparticles as contrast agents for computed tomography and photoacoustic imaging. Biomaterials.

[32] Chu Z, Chen L, Wang X, Yang Q, Zhao Q, Huang C (2019). Ultrasmall Au-Ag alloy nanoparticles: protein-directed synthesis, biocompatibility, and X-ray computed tomography imaging. ACS Biomater Sci Eng.

[33] Guo Z, Yang L, Chen M, Wen X, Liu H, Li J (2020). Molecular imaging of advanced atherosclerotic plaques with folate receptor-targeted 2D nanoprobes. Nano Res.

[34] Naha PC, Al Zaki A, Hecht E, Chorny M, Chhour P, Blankemeyer E (2014). Dextran coated bismuth-iron oxide nanohybrid contrast agents for computed tomography and magnetic resonance imaging. J Mater Chem B.

[35] Nosrati H, Salehiabar M, Mozafari F, Charmi J, Erdoğan N, Ghaffarlou M (2022). Preparation and evaluation of bismuth sulfide and magnetite-based theranostic nanohybrid as drug carrier and dual MRI/CT contrast agent. Appl Organomet Chem.

[36] Hernández-Rivera M, Kumar I, Cho SY, Cheong BY, Pulikkathara MX, Moghaddam SE (2017). High-performance hybrid bismuth-carbon nanotube based contrast agent for X-ray CT imaging. ACS Appl Mater Interfaces.

[37] Koshevaya E, Nazarovskaia D, Simakov M, Belousov A, Morozov V, Gandalipov E (2020). Surfactant-free tantalum oxide nanoparticles: synthesis, colloidal properties, and application as a contrast agent for computed tomography. J Mater Chem B.

[38] Freedman JD, Lusic H, Snyder BD, Grinstaff MW (2014). Tantalum oxide nanoparticles for the imaging of articular cartilage using X-ray computed tomography: visualization of ex vivo/in vivo murine tibia and ex vivo human index finger cartilage. Angew Chem Int Ed.

[39] Kim DH, Park JC, Jeon GE, Kim CS, Seo JH (2017). Effect of the size and shape of silver nanoparticles on bacterial growth and metabolism by monitoring optical density and fluorescence intensity. Biotechnol Bioprocess Eng.

[40] Abdelhalim MAK, Jarrar BM (2012). Histological alterations in the liver of rats induced by different gold nanoparticle sizes, doses and exposure duration. J Nanobiotechnol.

[41] Kim J-Y, Ryu JH, Schellingerhout D, Sun I-C, Lee S-K, Jeon S (2015). Direct imaging of cerebral thromboemboli using computed tomography and fibrin-targeted gold nanoparticles. Theranostics.

[42] Pei S, Sun Y, Fan D, Deng S, Mei H, Wang H (2021). Computed tomography imaging agent based on gold nanoparticles for internal iliac artery embolization after endovascular abdominal aortic repair and CCN3 protection mechanism. J Nanomater.

[43] Zhu W, Liang S, Wang J, Yang Z, Zhang L, Yuan T (2017). Europium-phenolic network coated BaGdF_5_ nanocomposites for tri-modal computed tomography/magnetic resonance/luminescence imaging. J Mater Sci: Mater Med.

[44] Toczek J, Boodagh P, Sanzida N, Ghim M, Salarian M, Gona K (2021). Computed tomography imaging of macrophage phagocytic activity in abdominal aortic aneurysm. Theranostics.

[45] Ter-Pogossian MM, Phelps ME, Hoffman EJ, Mullani NA (1975). A positron-emission transaxial tomograph for nuclear imaging (PETT). Radiology.

[46] Muehllehner G, Karp JS (2006). Positron emission tomography. Phys Med Biol.

[47] Bengel FM, Higuchi T, Javadi MS, Lautamäki R (2009). Cardiac positron emission tomography. J Am Coll Cardiol.

[48] Shen S, Li H, Ge S, Huang H, Zhang H, Li F (2021). ^18^F-fluorodeoxyglucose positron emission tomography for the detection of inflammatory lesions of the arterial vessel walls in Wistar rats. Exp Ther Med.

[49] Barwick TD, Lyons O, Mikhaeel N, Waltham M, O'Doherty M (2014). ^18^F-FDG PET-CT uptake is a feature of both normal diameter and aneurysmal aortic wall and is not related to aneurysm size. Eur J Nucl Med Mol Imaging.

[50] Alavi A, Werner TJ, Raynor W, Høilund-Carlsen PF, Revheim M-E (2021). Critical review of PET imaging for detection and characterization of the atherosclerotic plaques with emphasis on limitations of FDG-PET compared to NaF-PET in this setting. Am J Nucl Med Mol Imaging.

[51] Celeng C, de Keizer B, Merkely B, de Jong P, Leiner T, Takx RA (2018). PET molecular targets and near-infrared fluorescence imaging of atherosclerosis. Curr Cardiol Rep.

[52] Jeong HJ, Yoo RJ, Kim JK, Kim MH, Park SH, Kim H (2019). Macrophage cell tracking PET imaging using mesoporous silica nanoparticles via in vivo bioorthogonal F-18 labeling. Biomaterials.

[53] Kim EJ, Kim S, Seo HS, Lee YJ, Eo JS, Jeong JM (2016). Novel PET imaging of atherosclerosis with ^68^Ga-labeled NOTA-neomannosylated human serum albumin. J Nucl Med.

[54] Hultén LM, Levin M (2009). The role of hypoxia in atherosclerosis. Curr Opin Lipidol.

[55] Marsch E, Sluimer JC, Daemen MJ (2013). Hypoxia in atherosclerosis and inflammation. Curr Opin Lipidol.

[56] Savransky V, Nanayakkara A, Li J, Bevans S, Smith PL, Rodriguez A (2007). Chronic intermittent hypoxia induces atherosclerosis. Am J Respir Crit Care Med.

[57] Nie X, Randolph GJ, Elvington A, Bandara N, Zheleznyak A, Gropler RJ (2016). Imaging of hypoxia in mouse atherosclerotic plaques with ^64^Cu-ATSM. Nucl Med Biol.

[58] Toole BP (2009). Hyaluronan-CD44 interactions in cancer: paradoxes and possibilities. Clin Cancer Res.

[59] Beldman TJ, Senders ML, Alaarg A, Pérez-Medina C, Tang J, Zhao Y (2017). Hyaluronan nanoparticles selectively target plaque-associated macrophages and improve plaque stability in atherosclerosis. Acs Nano.

[60] Fischer JW (2019). Role of hyaluronan in atherosclerosis: current knowledge and open questions. Matrix Biol.

[61] Kantak MN, Bharate SS (2022). Analysis of clinical trials on biomaterial and therapeutic applications of chitosan: a review. Carbohydr Polym.

[62] Aibani N, Rai R, Patel P, Cuddihy G, Wasan EK (2021). Chitosan nanoparticles at the biological interface: implications for drug delivery. Pharmaceutics.

[63] Abd-Allah H, Abdel-Aziz RT, Nasr M (2020). Chitosan nanoparticles making their way to clinical practice: a feasibility study on their topical use for acne treatment. Int J Biol Macromol.

[64] Fairclough M, Ellis B, Boutin H, Jones A, McMahon A, Alzabin S (2017). Development of a method for the preparation of zirconium-89 radiolabelled chitosan nanoparticles as an application for leukocyte trafficking with positron emission tomography. Appl Radiat Isot.

[65] Poels K, van Leent MM, Reiche ME, Kusters PJ, Huveneers S, de Winther MP (2020). Antibody-mediated inhibition of CTLA4 aggravates atherosclerotic plaque inflammation and progression in hyperlipidemic mice. Cells.

[66] Jarr K-U, Ye J, Kojima Y, Nanda V, Flores AM, Tsantilas P (2020). ^18^F-fluorodeoxyglucose-positron emission tomography imaging detects response to therapeutic intervention and plaque vulnerability in a murine model of advanced atherosclerotic disease-brief report. Arterioscler Thromb Vasc Biol.

[67] Fiz F, Morbelli S, Piccardo A, Bauckneht M, Ferrarazzo G, Pestarino E (2015). ^18^F-NaF uptake by atherosclerotic plaque on PET/CT imaging: inverse correlation between calcification density and mineral metabolic activity. J Nucl Med.

[68] Bala G, Blykers A, Xavier C, Descamps B, Broisat A, Ghezzi C (2016). Targeting of vascular cell adhesion molecule-1 by ^18^F-labelled nanobodies for PET/CT imaging of inflamed atherosclerotic plaques. Eur Heart J Cardiovasc Imaging.

[69] Keliher EJ, Ye Y-X, Wojtkiewicz GR, Aguirre AD, Tricot B, Senders ML (2017). Polyglucose nanoparticles with renal elimination and macrophage avidity facilitate PET imaging in ischaemic heart disease. Nat Commun.

[70] Liu Y, Jiang Z, Yang X, Wang Y, Yang B, Fu Q (2024). Engineering nanoplatforms for theranostics of atherosclerotic plaques. Adv Healthcare Mater.

[71] Li X-G, Hagert C, Siitonen R, Virtanen H, Sareila O, Liljenback H (2016). ^18^F-labeling of mannan for inflammation research with positron emission tomography. ACS Med Chem Lett.

[72] Liu Y, Luehmann HP, Detering L, Pressly ED, McGrath AJ, Sultan D (2019). Assessment of targeted nanoparticle assemblies for atherosclerosis imaging with positron emission tomography and potential for clinical translation. ACS Appl Mater Interfaces.

[73] Sultan D, Li W, Detering L, Heo GS, Luehmann HP, Kreisel D (2021). Assessment of ultrasmall nanocluster for early and accurate detection of atherosclerosis using positron emission tomography/computed tomography. Nanomedicine.

[74] Nahrendorf M, Hoyer FF, Meerwaldt AE, van Leent MM, Senders ML, Calcagno C (2020). Imaging cardiovascular and lung macrophages with the positron emission tomography sensor ^64^Cu-macrin in mice, rabbits, and pigs. Circ Cardiovasc Imaging.

[75] Lobatto ME, Binderup T, Robson PM, Giesen LF, Calcagno C, Witjes J (2019). Multimodal positron emission tomography imaging to quantify uptake of ^89^Zr-labeled liposomes in the atherosclerotic vessel wall. Bioconjugateate Chem.

[76] Lee S-P, Im H-J, Kang S, Chung S-J, Cho YS, Kang H (2017). Noninvasive imaging of myocardial inflammation in myocarditis using ^68^Ga-tagged mannosylated human serum albumin positron emission tomography. Theranostics.

[77] Izquierdo-Garcia D, Diyabalanage H, Ramsay IA, Rotile NJ, Mauskapf A, Choi J-K (2022). Imaging high-risk atherothrombosis using a novel fibrin-binding positron emission tomography probe. Stroke.

[78] Lee SB, Lee HW, Singh TD, Li Y, Kim SK, Cho SJ (2017). Visualization of macrophage recruitment to inflammation lesions using highly sensitive and stable radionuclide-embedded gold nanoparticles as a nuclear bio-imaging platform. Theranostics.

[79] Van Geuns R-JM, Wielopolski PA, de Bruin HG, Rensing BJ, van Ooijen PM, Hulshoff M (1999). Basic principles of magnetic resonance imaging. Prog Cardiovasc Dis.

[80] Wood ML, Hardy PA (1993). Proton relaxation enhancement. J Magn Reson Imaging.

[81] Carr D, Brown J, Bydder G, Weinmann H, Speck U, Thomas D (1984). Intravenous chelated gadolinium as a contrast agent in NMR imaging of cerebral tumours. Lancet.

[82] Li L, Wang J, Wu M, He Y, Zhang H, Xu G (2019). Macrophage-targeted and clearable glutathione-based MRI nanoprobes for atherosclerosis molecular imaging. J Nanopart Res.

[83] Fracassi A, Cao J, Yoshizawa-Sugata N, Tóth É, Archer C, Gröninger O (2020). LDL-mimetic lipid nanoparticles prepared by surface KAT ligation for in vivo MRI of atherosclerosis. Chem Sci.

[84] Yoo SP, Pineda F, Barrett JC, Poon C, Tirrell M, Chung EJ (2016). Gadolinium-functionalized peptide amphiphile micelles for multimodal imaging of atherosclerotic lesions. ACS Omega.

[85] Tarin C, Carril M, Martin-Ventura JL, Markuerkiaga I, Padro D, Llamas-Granda P (2015). Targeted gold-coated iron oxide nanoparticles for CD163 detection in atherosclerosis by MRI. Sci Rep.

[86] Kim CW, Hwang B-H, Moon H, Kang J, Park E-H, Ihm S-H (2021). In vivo MRI detection of intraplaque macrophages with biocompatible silica-coated iron oxide nanoparticles in murine atherosclerosis. J Appl Biomater Funct Mater.

[87] Huang X, Lin C, Luo C, Guo Y, Li J, Wang Y (2021). Fe_3_O_4_@M nanoparticles for MRI-targeted detection in the early lesions of atherosclerosis. Nanomedicine.

[88] Kim M, Sahu A, Kim GB, Nam GH, Um W, Shin SJ (2018). Comparison of in vivo targeting ability between cRGD and collagen-targeting peptide conjugated nano-carriers for atherosclerosis. J Controlled Release.

[89] Bonnet S, Prévot G, Mornet S, Jacobin-Valat M-J, Mousli Y, Hemadou A (2021). A nano-emulsion platform functionalized with a fully human scFv-Fc antibody for atheroma targeting: towards a theranostic approach to atherosclerosis. Int J Mol Sci.

[90] Zhang S, Xu W, Gao P, Chen W, Zhou Q (2020). Construction of dual nanomedicines for the imaging and alleviation of atherosclerosis. Artif Cells Nanomed Biotechnol.

[91] Yao Y, Li B, Fu C, Teng G, Ma G, Liu N (2017). Anti-connective tissue growth factor detects and reduces plaque inflammation in early-stage carotid atherosclerotic lesions. Nanomedicine.

[92] Fan W-H, Pech M, Karnovsky MJ (2000). Connective tissue growth factor (CTGF) stimulates vascular smooth muscle cell growth and migration in vitro. Eur J Cell Biol.

[93] Wu Y, Zhang R, Tran HD, Kurniawan ND, Moonshi SS, Whittaker AK (2021). Chitosan nanococktails containing both ceria and superparamagnetic iron oxide nanoparticles for reactive oxygen species-related theranostics. ACS Appl Nano Mater.

[94] Hossaini Nasr S, Tonson A, El-Dakdouki MH, Zhu DC, Agnew D, Wiseman R (2018). Effects of nanoprobe morphology on cellular binding and inflammatory responses: hyaluronan-conjugated magnetic nanoworms for magnetic resonance imaging of atherosclerotic plaques. ACS Appl Mater Interfaces.

[95] Sherin S, Balachandran S, Abraham A (2020). Curcumin incorporated titanium dioxide nanoparticles as MRI contrasting agent for early diagnosis of atherosclerosis-rat model. Vet Anim Sci.

[96] Yao J, Yang Z, Huang L, Yang C, Wang J, Cao Y (2021). Low-intensity focused ultrasound-responsive ferrite-encapsulated nanoparticles for atherosclerotic plaque neovascularization theranostics. Adv Sci.

[97] Maiseyeu A, Di L, Ravodina A, Barajas-Espinosa A, Sakamoto A, Chaplin A (2022). Plaque-targeted, proteolysis-resistant, activatable and MRI-visible nano-GLP-1 receptor agonist targets smooth muscle cell differentiation in atherosclerosis. Theranostics.

[98] Woodside DG, Tanifum EA, Ghaghada KB, Biediger RJ, Caivano AR, Starosolski ZA (2018). Magnetic resonance imaging of atherosclerotic plaque at clinically relevant field strengths (1T) by targeting the integrin α4β1. Sci Rep.

[99] Shen ZT, Zheng S, Gounis MJ, Sigalov AB (2015). Diagnostic magnetic resonance imaging of atherosclerosis in apolipoprotein E knockout mouse model using macrophage-targeted gadolinium-containing synthetic lipopeptide nanoparticles. PLoS One.

[100] Bagalkot V, Badgeley MA, Kampfrath T, Deiuliis JA, Rajagopalan S, Maiseyeu A (2015). Hybrid nanoparticles improve targeting to inflammatory macrophages through phagocytic signals. J Controlled Release.

[101] Yu M, Ortega CA, Si K, Molinaro R, Schoen FJ, Leitao RF (2018). Nanoparticles targeting extra domain B of fibronectin-specific to the atherosclerotic lesion types Ⅲ, Ⅳ, and Ⅴ-enhance plaque detection and cargo delivery. Theranostics.

[102] Cheng D, Li X, Zhang C, Tan H, Wang C, Pang L (2015). Detection of vulnerable atherosclerosis plaques with a dual-modal single-photon-emission computed tomography/magnetic resonance imaging probe targeting apoptotic macrophages. ACS Appl Mater Interfaces.

[103] Mo H, Fu C, Wu Z, Liu P, Wen Z, Hong Q (2020). IL-6-targeted ultrasmall superparamagnetic iron oxide nanoparticles for optimized MRI detection of atherosclerotic vulnerable plaques in rabbits. RSC Adv.

[104] Prévot G, Kauss T, Lorenzato C, Gaubert A, Larivière M, Baillet J (2017). Iron oxide core oil-in-water nanoemulsion as tracer for atherosclerosis MPI and MRI imaging. Int J Pharm.

[105] Poon C, Gallo J, Joo J, Chang T, Bañobre-López M, Chung EJ (2018). Hybrid, metal oxide-peptide amphiphile micelles for molecular magnetic resonance imaging of atherosclerosis. J Nanobiotechnol.

[106] Wang Y, Chen J, Yang B, Qiao H, Gao L, Su T (2016). In vivo MR and fluorescence dual-modality imaging of atherosclerosis characteristics in mice using profilin-1 targeted magnetic nanoparticles. Theranostics.

[107] Kao C-W, Wu P-T, Liao M-Y, Chung I-J, Yang K-C, Tseng W-YI (2018). Magnetic nanoparticles conjugated with peptides derived from monocyte chemoattractant protein-1 as a tool for targeting atherosclerosis. Pharmaceutics.

[108] Qiao H, Wang Y, Zhang R, Gao Q, Liang X, Gao L (2017). MRI/optical dual-modality imaging of vulnerable atherosclerotic plaque with an osteopontin-targeted probe based on Fe_3_O_4_ nanoparticles. Biomaterials.

[109] Ye M, Zhou J, Zhong Y, Xu J, Hou J, Wang X (2019). SR-A-targeted phase-transition nanoparticles for the detection and treatment of atherosclerotic vulnerable plaques. ACS Appl Mater Interfaces.

[110] Xu W, Zhang S, Zhou Q, Chen W (2019). VHPKQHR peptide modified magnetic mesoporous nanoparticles for MRI detection of atherosclerosis lesions. Artif Cells Nanomed Biotechnol.

[111] Wu M, Li X, Guo Q, Li J, Xu G, Li G (2021). Magnetic mesoporous silica nanoparticles-aided dual MR/NIRF imaging to identify macrophage enrichment in atherosclerotic plaques. Nanomedicine.

[112] Ta HT, Li Z, Hagemeyer CE, Cowin G, Zhang S, Palasubramaniam J (2017). Molecular imaging of activated platelets via antibody-targeted ultra-small iron oxide nanoparticles displaying unique dual MRI contrast. Biomaterials.

[113] Jacobin-Valat M-J, Laroche-Traineau J, Larivière M, Mornet S, Sanchez S, Biran M (2015). Nanoparticles functionalised with an anti-platelet human antibody for in vivo detection of atherosclerotic plaque by magnetic resonance imaging. Nanomedicine.

[114] Zhang L, Tian XY, Chan CK, Bai Q, Cheng CK, Chen FM (2018). Promoting the delivery of nanoparticles to atherosclerotic plaques by DNA coating. ACS Appl Mater Interfaces.

[115] Wei H, Tan T, Cheng L, Liu J, Song H, Li L (2020). MRI tracing of ultrasmall superparamagnetic iron oxide nanoparticle-labeled endothelial progenitor cells for repairing atherosclerotic vessels in rabbits. Mol Med Rep.

[116] Banik B, Surnar B, Askins BW, Banerjee M, Dhar S (2019). Dual-targeted synthetic nanoparticles for cardiovascular diseases. ACS Appl Mater Interfaces.

[117] Larivière M, Lorenzato CS, Adumeau L, Bonnet S, Hémadou A, Jacobin-Valat M-J (2019). Multimodal molecular imaging of atherosclerosis: Nanoparticles functionalized with scFv fragments of an anti-αⅡbβ3 antibody. Nanomedicine.

[118] Mu D, Wang W, Li J, Lv P, Liu R, Tan Y (2021). Ultrasmall Fe (Ⅲ)-tannic acid nanoparticles to prevent progression of atherosclerotic plaques. ACS Appl Mater Interfaces.

[119] Mog B, Asase C, Chaplin A, Gao H, Rajagopalan S, Maiseyeu A (2019). Nano-antagonist alleviates inflammation and allows for MRI of atherosclerosis. Nanotheranostics.

[120] Suzuki M, Bachelet-Violette L, Rouzet F, Beilvert A, Autret G, Maire M (2015). Ultrasmall superparamagnetic iron oxide nanoparticles coated with fucoidan for molecular MRI of intraluminal thrombus. Nanomedicine.

[121] Segers FM, Ruder AV, Westra MM, Lammers T, Dadfar SM, Roemhild K (2022). MRI contrast-enhancement with superparamagnetic iron oxide nanoparticles amplify macrophage foam cell apoptosis in human and murine atherosclerosis. Cardiovasc. Res.

[122] Evans RJ, Lavin B, Phinikaridou A, Chooi KY, Mohri Z, Wong E (2020). Targeted molecular iron oxide contrast agents for imaging atherosclerotic plaque. Nanotheranostics.

[123] Zhang L, Xue S, Ren F, Huang S, Zhou R, Wang Y (2021). An atherosclerotic plaque-targeted single-chain antibody for MR/NIR-Ⅱimaging of atherosclerosis and anti-atherosclerosis therapy. J Nanobiotechnol.

[124] Wang Y, Zhang Y, Wang Z, Zhang J, Qiao RR, Xu M (2019). Optical/MRI dual-modality imaging of M1 macrophage polarization in atherosclerotic plaque with MARCO-targeted upconversion luminescence probe. Biomaterials.

[125] Wei X, Ying M, Dehaini D, Su Y, Kroll AV, Zhou J (2018). Nanoparticle functionalization with platelet membrane enables multifactored biological targeting and detection of atherosclerosis. ACS Nano.

[126] Cismaru G, Serban T, Tirpe A (2021). Ultrasound methods in the evaluation of atherosclerosis: from pathophysiology to clinic. Biomedicines.

[127] Henein MY, Vancheri S, Bajraktari G, Vancheri F (2020). Coronary atherosclerosis imaging. Diagnostics.

[128] Iannuzzi A, Rubba P, Gentile M, Mallardo V, Calcaterra I, Bresciani A (2021). Carotid atherosclerosis, ultrasound and lipoproteins. Biomedicines.

[129] Cuspidi C, Lonati L, Sampieri L, Pelizzoli S, Pontiggia G, Leonetti G (1996). Left ventricular concentric remodelling and carotid structural changes in essential hypertension. J Hypertens.

[130] Gramiak R, Shah PM (1968). Echocardiography of the aortic root. Invest Radiol.

[131] Yang H, Sun Y, Wei J, Xu L, Tang Y, Yang L (2019). The effects of ultrasound-targeted microbubble destruction (UTMD) carrying IL-8 monoclonal antibody on the inflammatory responses and stability of atherosclerotic plaques. Biomed Pharmacother.

[132] Zhang X, Wu M, Zhang Y, Zhang J, Su J, Yang C (2020). Molecular imaging of atherosclerotic plaque with lipid nanobubbles as targeted ultrasound contrast agents. Colloids Surf, B.

[133] Li S, Gou T, Wang Q, Chen M, Chen Z, Xu M (2020). Ultrasound/optical dual-modality imaging for evaluation of vulnerable atherosclerotic plaques with osteopontin targeted nanoparticles. Macromol Biosci.

[134] Moccetti F, Weinkauf CC, Davidson BP, Belcik JT, Marinelli ER, Unger E (2018). Ultrasound molecular imaging of atherosclerosis using small-peptide targeting ligands against endothelial markers of inflammation and oxidative stress. Ultrasound Med Biol.

[135] Yan F, Sun Y, Mao Y, Wu M, Deng Z, Li S (2018). Ultrasound molecular imaging of atherosclerosis for early diagnosis and therapeutic evaluation through leucocyte-like multiple targeted microbubbles. Theranostics.

[136] Mehta S, Bongcaron V, Nguyen TK, Jirwanka Y, Maluenda A, Walsh AP (2022). An ultrasound-responsive theranostic cyclodextrin-loaded nanoparticle for multimodal imaging and therapy for atherosclerosis. Small.

[137] Gawaz M, Langer H, May AE (2005). Platelets in inflammation and atherogenesis. J Clin Invest.

[138] Hu X, Zhao P, Zhang J, Zhu Y, Zhou W, Hong K (2023). Ultrasound-assisted biomimetic nanobubbles for targeted treatment of atherosclerosis. Nanomedicine.

[139] Zhou W, Chen D, Li K, Yuan Z, Chen X (2023). Multimodal photoacoustic imaging in analytic vulnerability of atherosclerosis. iRADIOLOGY.

[140] Manwar R, Zafar M, Xu Q (2020). Signal and image processing in biomedical photoacoustic imaging: a review. Optics.

[141] Choi W, Park B, Choi S, Oh D, Kim J, Kim C (2023). Recent advances in contrast-enhanced photoacoustic imaging: overcoming the physical and practical challenges. Chem Rev.

[142] Mantri Y, Jokerst JV (2020). Engineering plasmonic nanoparticles for enhanced photoacoustic imaging. ACS Nano.

[143] Steinbrueck A, Karges J (2023). Metal complexes and nanoparticles for photoacoustic imaging. ChemBioChem.

[144] Guo T, Tang Q, Guo Y, Qiu H, Dai J, Xing C (2020). Boron quantum dots for photoacoustic imaging-guided photothermal therapy. ACS Appl Mater Interfaces.

[145] Bodian S, Colchester RJ, Macdonald TJ, Ambroz F, Briceno de Gutierrez M, Mathews SJ (2021). CuInS_2_ quantum dot and polydimethylsiloxane nanocomposites for all-optical ultrasound and photoacoustic imaging. Adv Mater Interfaces.

[146] Chen SH, Liu H, Huang B, Zheng J, Zhang ZL, Pang DW (2024). Biosynthesis of NIR-Ⅱ Ag_2_Se quantum dots with bacterial catalase for photoacoustic imaging and alleviating-hypoxia photothermal therapy. Small.

[147] Zhao Y, Song M, Yang X, Yang J, Du C, Wang G (2020). Amorphous Ag_2-x_Cu_x_S quantum dots:“all-in-one” theranostic nanomedicines for near-infrared fluorescence/photoacoustics dual-modal-imaging-guided photothermal therapy. Chem Eng J.

[148] Li C, Liu C, Fan Y, Ma X, Zhan Y, Lu X (2021). Recent development of near-infrared photoacoustic probes based on small-molecule organic dye. RSC Chem Biol.

[149] Wang Y, Liu X, Liu C, Su Y, Cong H, Gong S (2023). Design and preparation of organic small molecules in NIR-Ⅱ and their application in fluorescence imaging/photothermal therapy/photoacoustic imaging. Dyes Pigm.

[150] Li Y, Zhou H, Bi R, Li X, Zha M, Yang Y (2021). Acceptor engineering of small-molecule fluorophores for NIR-Ⅱ fluorescence and photoacoustic imaging. J Mater Chem B.

[151] Men X, Wang F, Chen H, Liu Y, Men X, Yuan Y (2020). Ultrasmall semiconducting polymer dots with rapid clearance for second near-infrared photoacoustic imaging and photothermal cancer therapy. Adv Funct Mater.

[152] Li J, Li S, Yang S, Liang M, Jiang X, Wu W (2022). Semiconductor polymer with strong NIR-Ⅱ absorption for photoacoustic imaging and photothermal therapy. ACS Appl Bio Mater.

[153] Ong SY, Zhang C, Dong X, Yao SQ (2021). Recent advances in polymeric nanoparticles for enhanced fluorescence and photoacoustic imaging. Angew Chem Int Ed.

[154] Ma Y, Xu L, Yin B, Shang J, Chen F, Xu J (2021). Ratiometric semiconducting polymer nanoparticle for reliable photoacoustic imaging of pneumonia-induced vulnerable atherosclerotic plaque in vivo. Nano Lett.

[155] Xu H, She P, Zhao Z, Ma B, Li G, Wang Y (2023). Duplex responsive nanoplatform with cascade targeting for atherosclerosis photoacoustic diagnosis and multichannel combination therapy. Adv Mater.

[156] Ma Y, Shang J, Liu L, Li M, Xu X, Cao H (2023). Rational design of a double-locked photoacoustic probe for precise in vivo imaging of cathepsin B in atherosclerotic plaques. J Am Chem Soc.

[157] Ma B, Chen Z, Xu H, Sun S, Huang C, Li G (2024). Biomimetic Targeting Nanoplatform for Atherosclerosis Theranostics Via Photoacoustic Diagnosis and “Hand-In-Hand” Immunoregulation. Adv Funct Mater.

[158] Gao W, Li X, Liu Z, Fu W, Sun Y, Cao W (2018). A redox-responsive self-assembled nanoprobe for photoacoustic inflammation imaging to assess atherosclerotic plaque vulnerability. Anal Chem.

[159] Ge X, Cui H, Kong J, Lu SY, Zhan R, Gao J (2020). A non-invasive nanoprobe for in vivo photoacoustic imaging of vulnerable atherosclerotic plaque. Adv Mater.

[160] Lee D-E, Koo H, Sun I-C, Ryu JH, Kim K, Kwon IC (2012). Multifunctional nanoparticles for multimodal imaging and theragnosis. Chem Soc Rev.

[161] Louie A (2010). Multimodality imaging probes: design and challenges. Chem Rev.

[162] Vandenberghe S, Marsden PK (2015). PET-MRI: a review of challenges and solutions in the development of integrated multimodality imaging. Phys Med Biol.

[163] Nikolaou K, Poon M, Sirol M, Becker CR, Fayad ZA (2003). Complementary results of computed tomography and magnetic resonance imaging of the heart and coronary arteries: a review and future outlook. Cardiol Clin.

[164] Ni D, Zhang J, Bu W, Zhang C, Yao Z, Xing H (2016). PEGylated NaHoF_4_ nanoparticles as contrast agents for both X-ray computed tomography and ultra-high field magnetic resonance imaging. Biomaterials.

[165] Tong W, Hui H, Shang W, Zhang Y, Tian F, Ma Q (2021). Highly sensitive magnetic particle imaging of vulnerable atherosclerotic plaque with active myeloperoxidase-targeted nanoparticles. Theranostics.

[166] Zhang P, Li L, Lin L, Shi J, Wang LV (2019). In vivo superresolution photoacoustic computed tomography by localization of single dyed droplets. Light: Sci Appl.

[167] Liu X, Gao R, Chen C, Li X, Yu C, Chen Y (2022). Noninvasive photoacoustic computed tomography/ultrasound imaging to identify high-risk atherosclerotic plaques. Eur J Nucl Med Mol Imaging.

[168] Xie Z, Yang Y, He Y, Shu C, Chen D, Zhang J (2020). In vivo assessment of inflammation in carotid atherosclerosis by noninvasive photoacoustic imaging. Theranostics.

[169] Meester EJ, Krenning B, De Swart J, Segbers M, Barrett H, Bernsen M (2019). Perspectives on small animal radionuclide imaging; considerations and advances in atherosclerosis. Front Med.

[170] Seo Y, Mari C, Hasegawa BH (2008). Technological development and advances in single-photon emission computed tomography/computed tomography. Semin Nucl Med.

[171] Li X, Wang C, Tan H, Cheng L, Liu G, Yang Y (2016). Gold nanoparticles-based SPECT/CT imaging probe targeting for vulnerable atherosclerosis plaques. Biomaterials.

[172] Ma Y, Ma Y, Gao M, Han Z, Jiang W, Gu Y (2021). Platelet-mimicking therapeutic system for noninvasive mitigation of the progression of atherosclerotic plaques. Adv Sci.

[173] Liu G, Hu Y, Xiao J, Li X, Li Y, Tan H (2016). ^99m^Tc-labelled anti-CD11b SPECT/CT imaging allows detection of plaque destabilization tightly linked to inflammation. Sci Rep.

[174] Luehmann HP, Detering L, Fors BP, Pressly ED, Woodard PK, Randolph GJ (2016). PET/CT imaging of chemokine receptors in inflammatory atherosclerosis using targeted nanoparticles. J Nucl Med.

[175] Senders ML, Hernot S, Carlucci G, Voort JCvd, Fay F, Calcagno C (2019). Nanobody-facilitated multiparametric PET/MRI phenotyping of atherosclerosis. JACC Cardiovasc Imaging.

[176] Pellico J, Fernández-Barahona I, Ruiz-Cabello Js, Gutiérrez L, Muñoz-Hernando M, Sánchez-Guisado MJ (2021). HAP-multitag, a PET and positive MRI contrast nanotracer for the longitudinal characterization of vascular calcifications in atherosclerosis. ACS Appl Mater Interfaces.

[177] Chen J, Yang X-Q, Qin M-Y, Zhang X-S, Xuan Y, Zhao Y-D (2015). Hybrid nanoprobes of bismuth sulfide nanoparticles and CdSe/ZnS quantum dots for mouse computed tomography/fluorescence dual mode imaging. J Nanobiotechnol.

[178] Townsend DW (2008). Multimodality imaging of structure and function. Phys Med Biol.

[179] Li Y, Pan Y, Wu X, Li Y, Wang H, Zhu H (2019). Dual-modality imaging of atherosclerotic plaques using ultrasmall superparamagnetic iron oxide labeled with rhodamine. Nanomedicine.

[180] Zan C, An J, Wu Z, Li S (2023). Engineering molecular nanoprobes to target early atherosclerosis: precise diagnostic tools and promising therapeutic carriers. Nanotheranostics.

[181] Lameijer M, Binderup T, Van Leent MM, Senders ML, Fay F, Malkus J (2018). Efficacy and safety assessment of a TRAF6-targeted nanoimmunotherapy in atherosclerotic mice and non-human primates. Nat Biomed Eng.

[182] Lee WH, Rho JG, Yang Y, Lee S, Kweon S, Kim H-M (2022). Hyaluronic acid nanoparticles as a topical agent for treating psoriasis. ACS Nano.

[183] Su T, Wang Y-B, Han D, Wang J, Qi S, Gao L (2017). Multimodality imaging of angiogenesis in a rabbit atherosclerotic model by GEBP11 peptide targeted nanoparticles. Theranostics.

[184] Liu G, Hu Y, Xiao J, Li X, Li Y, Tan H (2016). ^99m^Tc-labelled anti-CD11b SPECT/CT imaging allows detection of plaque destabilization tightly linked to inflammation. Sci Rep.

[185] Hu Y, Liu G, Zhang H, Li Y, Gray BD, Pak KY (2018). A comparison of [99m Tc] duramycin and [99m Tc] annexin V in SPECT/CT imaging atherosclerotic plaques. Mol Imaging Biol.

[186] Meester E, Krenning B, De Blois R, Norenberg J, de Jong M, Bernsen M (2019). Imaging of atherosclerosis, targeting LFA-1 on inflammatory cells with ^111^In-DANBIRT. J Nucl Cardiol.

[187] Liang M, Tan H, Zhou J, Wang T, Duan D, Fan K (2018). Bioengineered H-ferritin nanocages for quantitative imaging of vulnerable plaques in atherosclerosis. ACS Nano.

[188] Varasteh Z, Hyafil F, Anizan N, Diallo D, Aid-Launais R, Mohanta S (2017). Targeting mannose receptor expression on macrophages in atherosclerotic plaques of apolipoprotein E-knockout mice using ^111^In-tilmanocept. EJNMMI Res.

[189] Tan H, Zhou J, Yang X, Abudupataer M, Li X, Hu Y (2017). ^99m^Tc-labeled bevacizumab for detecting atherosclerotic plaque linked to plaque neovascularization and monitoring antiangiogenic effects of atorvastatin treatment in ApoE^-/-^ mice. Sci Rep.

[190] Guan M, Zhu S, Li S (2021). Recent progress in nanomedicine for melanoma theranostics with emphasis on combination therapy. Front Bioeng Biotechnol.

[191] Younis MA, Tawfeek HM, Abdellatif AA, Abdel-Aleem JA, Harashima H (2022). Clinical translation of nanomedicines: Challenges, opportunities, and keys. Adv Drug Delivery Rev.

[192] Metselaar JM, Lammers T (2020). Challenges in nanomedicine clinical translation. Drug Delivery Transl Res.

[193] Zhang S, Liu Y, Cao Y, Zhang S, Sun J, Wang Y (2022). Targeting the Microenvironment of Vulnerable Atherosclerotic Plaques: An Emerging Diagnosis and Therapy Strategy for Atherosclerosis. Adv Mater.

[194] Liu Y, Yang G, Hui Y, Ranaweera S, Zhao CX (2022). Microfluidic nanoparticles for drug delivery. Small.

[195] Shepherd SJ, Issadore D, Mitchell MJ (2021). Microfluidic formulation of nanoparticles for biomedical applications. Biomaterials.

